# NK cells in hypoxic skin mediate a trade-off between wound healing and antibacterial defence

**DOI:** 10.1038/s41467-021-25065-w

**Published:** 2021-08-04

**Authors:** Michal Sobecki, Ewelina Krzywinska, Shunmugam Nagarajan, Annette Audigé, Khanh Huỳnh, Julian Zacharjasz, Julien Debbache, Yann Kerdiles, Dagmar Gotthardt, Norihiko Takeda, Joachim Fandrey, Lukas Sommer, Veronika Sexl, Christian Stockmann

**Affiliations:** 1 University of Zurich, Institute of Anatomy, Zurich, Switzerland; 2 Centre d’Immunologie de Marseille-Luminy, Aix Marseille Université UM2, Inserm, U1104, CNRS UMR7280, Marseille, France; 3 Institute of Pharmacology and Toxicology, University of Veterinary Medicine, Vienna, Austria; 4 Division of Cardiology and Metabolism, Center for Molecular Medicine, Jichi Medical University, 3311-1 Yakushiji, Tochigi, Japan; 5 Institut für Physiologie, Universitätsklinikum Essen, Universität Duisburg-Essen, Essen, Germany; 6 Comprehensive Cancer Center Zurich, Zurich, Switzerland

**Keywords:** Antimicrobial responses, Acute inflammation, Innate lymphoid cells, NK cells

## Abstract

During skin injury, immune response and repair mechanisms have to be coordinated for rapid skin regeneration and the prevention of microbial infections. Natural Killer (NK) cells infiltrate hypoxic skin lesions and Hypoxia-inducible transcription factors (HIFs) mediate adaptation to low oxygen. We demonstrate that mice lacking the Hypoxia-inducible factor (HIF)-1α isoform in NK cells show impaired release of the cytokines Interferon (IFN)-γ and Granulocyte Macrophage - Colony Stimulating Factor (GM-CSF) as part of a blunted immune response. This accelerates skin angiogenesis and wound healing. Despite rapid wound closure, bactericidal activity and the ability to restrict systemic bacterial infection are impaired. Conversely, forced activation of the HIF pathway supports cytokine release and NK cell-mediated antibacterial defence including direct killing of bacteria by NK cells despite delayed wound closure. Our results identify, HIF-1α in NK cells as a nexus that balances antimicrobial defence versus global repair in the skin.

## Abstract (web summary)

During wound healing and infection in the skin there is a hypoxic environment involving HIF-1α and NK cells. Here the authors show that NK cells through HIF-1α provide a cross-regulatory balance to provide an adequate antimicrobial defence that can inhibit subsequent wound healing.

## Introduction

After tissue injury, an adequate immune and repair response are prerequisites for rapid wound closure and the prevention of microbial infections^1^. Skin injury triggers key components of the skin repair and defence machinery including inflammation and regenerative angiogenesis, that have to be tightly coordinated^2^. Innate lymphoid cells (ILCs) are an early source of cytokines and particularly, NKp46^+^ group 1 ILCs (ILC1s), that comprise Natural Killer (NK) cells, guide inflammation and tailor the immune response to the type of the encountered insult^3^. Whereas the role of skin-resident ILC1s for steady-state skin homoeostasis is increasingly recognised, the significance of infiltrating NK cells for skin repair and antimicrobial defence remains unknown^4,5^. NK cells directly kill tumour cells and microbes^6,7^ and secrete cytokines including Interferon y (IFN-γ), Granulocyte Macrophage-Colony Stimulating Factor (GM-CSF) and Tumour Necrosis Factor (TNF) to instruct immune responses as well as repair processes^3,6,8,9^. IFN-γ and GM-CSF drive macrophage activation and favour proinflammatory M1 over a pro-regenerative M2 polarisation^1,2^. In addition, IFN-γ has been shown to exert anti-angiogenic effects^10^. Hypoxia is a characteristic feature of the tissue microenvironment during skin repair and bacterial infections, with tissue oxygen tensions lower than 10 mmHg in wounds and necrotic tissue foci^11,12^. Wound-infiltrating NK cells need to function under such conditions and adapt to low oxygen, which is mediated by Hypoxia-inducible transcription factors (HIFs), with HIF-1 and HIF-2 being the most extensively studied isoforms^13,14^. HIFs are basic-helix-loop-helix transcription factors that consist of a constitutively expressed β-subunit and an oxygen-regulated α-subunit that is hydroxylated by prolyl hydroxylases in the presence of oxygen and subsequently degraded through the ubiquitin proteasome pathway via interaction with its negative regulator von Hippel-Lindau (VHL) protein^15,16^. The role of HIF in tumour-infiltrating NK cells is controversial but in general evidence dominates that HIFs are important for NK cell performance in low oxygen environments^17,18^. Yet, the impact of the hypoxic response in NK cells upon skin injury and infection remains enigmatic. We here sought to elucidate if and how the HIF pathway in NKp46-expressing NK cells modulates wound healing and antimicrobial defence.

Here we show that hypoxic NK cells are a key element to balance antimicrobial defence versus repair mechanisms in the skin. Loss of HIF-1α in NK cells impairs release of IFN-γ and GM-CSF and the mounting of an adequate myeloid cell response. This results in accelerated wound healing but reduces the defence against bacterial skin infections. In conclusion, we propose that adequate antimicrobial defence in the skin is achieved at the expense of a limited repair capacity, whereas acceleration of physiological wound healing comes with a lower response against infections.

## Results

### Deletion of HIF-1α in NKp46^+^ cells accelerates skin wound healing

**Fig. 1:**
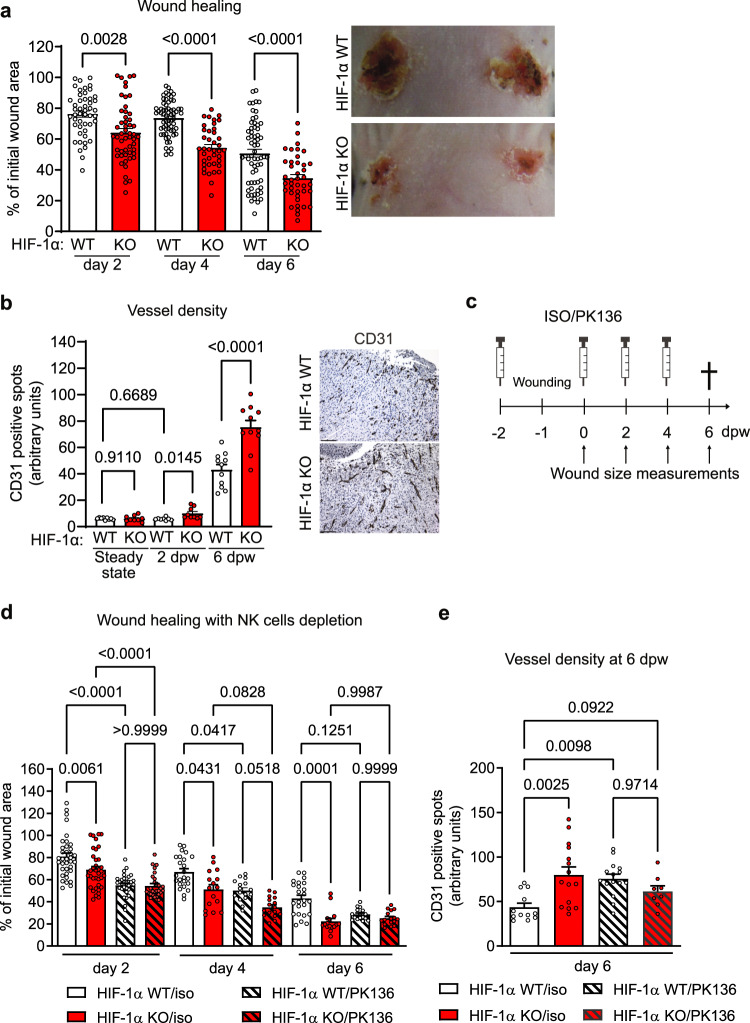
Genetic targeting of HIF-1α in NKp46^+^ cells accelerates wound healing. **a** Left, quantification of wound areas at 2, 4 and 6 days post wounding (dpw) in WT and HIF-1α KO animals in relation to their respective initial size at day 0. Pooled data of 4 experiments, (day 2, *n* = 48, WT and *n* = 52, HIF-1α KO, *p* = 0.0028; day 4 and 6, *n* = 64, WT and *n* = 40, HIF-1α KO, *p* < 0.0001), one-way ANOVA test. Right, macroscopic images of wounds from WT and HIF-1α KO animals, at 6 dpw. **b** Left, quantitative analysis of CD31-positive endothelial cells at steady state skin and in wounds at 2 and 6 dpw (steady state, *n* = 9; day 2, *n* = 11; day 6, *n* = 8) and HIF-1α KO (steady state, *n* = 9; day 2, *n* = 9, day 6, *n* = 9), pooled data of 3 experiments, one-way ANOVA test. Right, representative CD31, endothelial cell immunohistochemistry in skin wounds, scale bar, 100 μm. **c** Experimental layout for NK1.1^+^ cell depletion during wound healing. Control animals received i.p. injections of isotype control antibody. ISO - isotype control antibody; PK136 - NK1.1 antibody. **d** Quantification of wound areas at day 2, 4 and 6 pw in WT and HIF-1α KO animals with or without NK1.1+ cell depletion. Pooled data of 2 different experiments for 2 dpw (day 2, *n* = 36 for WT/iso, *n* = 32 for HIF-1α KO/iso, *n* = 36 for WT/PK136, *n* = 28 for HIF-1α KO/PK136; day 4 and 6, *n* = 24 for WT/iso, *n* = 16 for HIF-1α KO/iso, *n* = 16 for WT/PK136, *n* = 16 for HIF-1α KO/PK136), two-ways ANOVA test. **e** Quantitative analysis of CD31^+^ endothelial cells in skin wounds at 6 dpw from WT and HIF-1α KO animals with or without NK1.1^+^ cell depletion, (*n* = 12 for WT/iso, *n* = 15 for HIF-1α KO/iso, *n* = 14 for WT/PK136, *n* = 8 for HIF-1α KO/PK136), two-ways ANOVA test. Data are mean values ± SEM.

First, we performed immune phenotyping of the skin at steady state from mice with an in vivo, targeted deletion of HIF-1α in NK cells. The deletion was achieved by crosses of the loxP-flanked *Hif1a* allele^17^ to the *Ncr1* (NKp46) promoter-driven Cre recombinase^17^, specific to NKp46-expressing innate lymphoid cells, including NK cells (*Hif1α*^fl+/fl+/^*Ncr1*^cre+^ mice, termed HIF-1α KO). The skin of HIF-1α KO mice exhibited a decrease of NKp46+ cells in frequency and absolute number, whereas neutrophils, monocytes and macrophages were similarly abundant across genotypes (Supplementary Fig. 2a and Supplementary Fig. 1a, b for a detailed gating strategy). This suggests that HIF-1α supports migration or expansion of NK cells in the skin at steady state. Next, we tested the impact of HIF-1α on proliferation and migration of splenic NK cells. Interestingly, the absence of HIF-1α in NK cells leads to an in vitro proliferation defect at later stages of NK cell expansion as assessed by CFSE dilution in response to the cytokine stimulation IL-2 for five days, whereas migration towards the chemokine CCL5 is not affected (Supplementary Fig. 2b). To test the role of HIF-1α in NKp46^+^ cells during wound healing, we performed 6 mm circular punch biopsies on the back skin of WT and HIF-1α KO mice. Compared to their WT littermates, wound closure in HIF-1α KO mice was accelerated, which was observed as early as day 2 post-wounding (Fig. 1a). Neoformation of the microvasculature is crucial for sustained perfusion of the wound margins and determines the pace of wound closure^19^ and analysis of wound margins revealed the presence of vessel-associated NKp46-expressing cells (Supplementary Fig. 2c). The key role of NK cells in the fine-tuning of tumour angiogenesis^17^ prompted us to analyse the vascular density in skin wounds by quantifying CD31 positive blood vessels. Whereas, the skin vessel density is similar across genotypes at steady state (Fig. 1b), we found a pronounced and significant increase in capillary density in wound beds from HIF1α KO mice as early as day 2 post-wounding (Fig. 1b) that parallels the accelerated wound healing (Fig. 1a). The fact that the vessel density in in HIF-1α KO mice is already increased 48 h post-insult, whereas in WT mice the vascular density is similar to the steady state level, suggests that the process of angiogenesis is initiated earlier in the skin of HIF-1α KO mice than in WT mice.

The impact of NK cells on skin regeneration was investigated by antibody-mediated (anti-NK1.1. antibody PK136) NK cell depletion in WT and HIF-1α KO mice^17^ prior to wounding (Fig. 1c and Supplementary Fig. 2c). NK cell depletion resulted in a significantly accelerated wound closure in WT and HIF-1α KO mice as early as two days after wounding (Fig. 1d). At endpoint, this was associated with increased vessel density in HIF-1α KO mice and NK cell-depleted WT mice (Fig. 1e). These data led us to conclude that NK cell depletion phenocopies the changes induced by NK cell-specific HIF-1α loss to some extent. NK cells possess the ability to slow down wound healing and vascular remodelling in a HIF-1α-dependent manner.

### HIF-1α in NK cells controls the cytokine response and wound healing

**Fig. 2:**
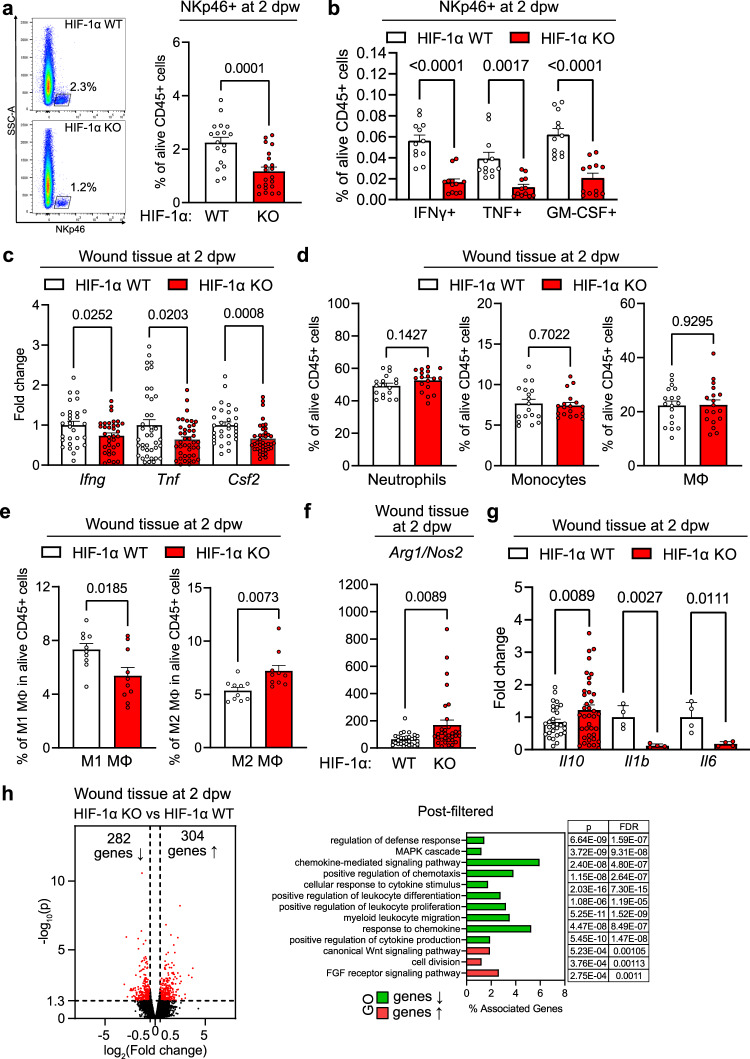
HIF-1α in NK cells controls the early cytokine response and skin regeneration. Analysis of skin wounds from WT and HIF-1α KO animals at 2 days post wounding (dpw). **a** Left, FACS plots showing the percentage of NKp46^+^ cells within CD45^+^ cells. Right, quantification of NKp46^+^ cells, pooled data of 4 experiments, (*n* = 18 mice for WT and *n* = 21 for HIF-1α KO), two-tailed Student’s *t* test. **b** FACS analysis of IFNγ-, TNF- and GM-CSF-expressing NKp46^+^NK1.1^+^ cells, pooled data of 3 experiments, (*n* = 12 mice), two-tailed Student’s *t* test. **c** Gene expression of *Ifng, Tnf* and *Csf2*, pooled data of 4 experiments, (*Ifng*, *n* = 30 wounds for WT and *n* = 32 for HIF-1α KO; *Tnf* *n* = 40 for WT and *n* = 40 for HIF-1α KO; *Csf2*, *n* = 30 for WT and *n* = 40 for HIF-1α KO), two-tailed Student’s *t* test. **d** FACS analysis for neutrophils (CD11b^+^, Ly6G^+^), monocytes (CD11b^+^, Ly6C^+^) and macrophages (CD11b^+^, Ly6C^+^, F4/80^+^), pooled data of 4 experiments, (n=18 mice), two-tailed Student’s t test. **e** FACS analysis for M1 (CD80^+^, MHCII^+^) and M2 macrophages (CD206^+^), pooled data of 3 experiments, (*n* = 10 mice), two-tailed Student’s *t* test. **f** Ratio of *Arg1* and Nos2 expression, pooled data of 4 experiments, (*n* = 28 wounds for WT and *n* = 32 for HIF-1α KO), two-tailed Student’s *t* test. **g** Gene expression for *Il10, Il1b* and *Il6*, pooled data of 4 experiments for *Il10* and 2 experiments for *Il1b* and *Il6*, (*Il10*, *n* = 30 wounds for WT and *n* = 38 for HIF-1α KO; *n* = 4 for *Il1b* and *Il6*), two-tailed Student’s *t* test. **h** Left, volcano plot for RNA sequencing analysis. Red data points meet the thresholds of log_2_(FC) above 0.5 and under −0.5, *P* < 0.05. Left, gene ontology (GO) prediction of the post filtered genes for the cytokines, chemokines and growth factors and corresponding receptors from the total pool of the significantly downregulated (282 genes) and upregulated genes (304 genes) with statistical readout (*p* value and FDR). Differential expression analysis was performed using the DESeq2 R package along with Wald test. Data are mean values ± SEM.

We wondered whether loss of HIF-1α affects infiltration and phenotype of wound-infiltrating NKp46^+^ cells (Fig. 1a, b). Flow cytometry on NKp46^+^ cells isolated from wound beds revealed a reduction of NKp46^+^ cells upon deletion of in HIF-1α KO (Fig. 2a and Supplementary Fig. 3a) at day 2 post wounding coinciding with first differences in wound closure. The vast majority of infiltrating NKp46^+^ cells (≈98%) were ILC1s (NKp46^+^, NK1.1^+^). Only few (≈2%) of ILC3s were present (NKp46^+^, NK1.1^−^, RORγt^+^) (Supplementary Fig. 3b and  1a for a detailed gating strategy). ILC1s can be further divided into NK cells (CD49b^+^ and Eomes^+^), non-NK cell ILC1s (CD49a^+^ and Tbet^+^) and intermediate ILC1s (CD49b^+^, CD49a^+^)^20^. The absence of HIF-1α during wound healing shifted the balance of intermediate ILC1s and ILC1s towards predominantly NK cells, which were generally the most abundant NKp46^+^ subset (≈82% and ≈89% of NKp46^+^, respectively) (Supplementary Fig. 3c, d). Loss of HIF-1α also impacted on NKp46^+^ cell function by modulating the production of the signature cytokines IFN-γ, GM-CSF and TNF. The frequency of wound-infiltrating IFN-γ^+^, GM-CSF^+^ and TNF^+^ NKp46^+^ cells was reduced (Fig. 2b and Supplementary Fig. 4a). Similarly, wound-associated HIF-1α null NKp46^+^ cells showed significantly lower levels of IFN-γ- and GM-CSF, whereas TNF expression was similar across genotypes (Supplementary Fig. 4b). This was further corroborated by experiments with purified splenic NK cells followed by in vitro stimulation with PMA/ionomycin, which showed reduced IFN-γ and GM-CSF expression in HIF-1α null NKp46^+^ cells (Supplementary Fig. 4c). Gene expression analysis of wound tissue confirmed the decrease in *Ifng, Tnf* and *Csf2* (the gene that encodes for GM-CSF) transcripts in HIF-1α KO mice (Fig. 2c). This indicates that NKp46^+^ cells significantly contribute to the overall cytokine pool at early stages of wound healing. The NK cell-derived soluble form of VEGF receptor 1 (sVEGFR1) binds and sequesters VEGF with high affinity, thereby reducing VEGF bioavailability and angiogenic signalling in the tumour microenvironment^17^. We detect a marked decrease in the expression of the angiostatic sVEGFR1 (encoded by the gene *sFlt1)* in wounds from HIF-1α KO mice indicating the dependence of *sFlt1* expression from HIF-1α. In contrast, VEGF expression was comparable across genotypes (Supplementary Fig. 5a). Loss of HIF-1α in wound-infiltrating NK cells thereby results in a decreased expression of two main antiangiogenic cytokines, IFN-γ and sFlt1. Together with the observation of increased angiogenesis during wound closure, this prompted us to address whether NK cells also regulate endothelial cell migration, which was explored in a classical in vitro scratch-wound assay. Supernatants derived from splenocytes after activation with the NK cell ligand NK1.1. in normoxia (20% O_2_) or hypoxia (2% O_2_) were added to a layer of endothelial cells after induction of a scratch. The supernatant of WT splenocytes substantially inhibited cell migration and wound closure (Supplementary Fig. 5b). In contrast, endothelial cell migration was unaffected by supernatants of HIF-1α KO splenocytes (Supplementary Fig. 5b). This suggests, that NK cells slow down angiogenesis and wound healing in a HIF-1α-dependent manner and that NK cell-derived cytokines are critical mediators of early skin regeneration.

Given the pivotal role of IFN-γ and GM-CSF in orchestrating the early innate immune response and macrophage polarisation^3,8,9,21^, we analysed the impact of HIF-1α in NK cells on the composition of wound-infiltrating immune cells. The frequency and absolute number of wound-associated neutrophils (CD11b^+^, Ly6G^+^), monocytes (CD11b^+^, Ly6G^low^, Ly6C^+^, F4/80^−^) and macrophages (CD11b^+^, Ly6G^low^, Ly6C^+^, F4/80^+^) was similar across genotypes (Fig. 2d, Supplementary Fig. 6 and see Supplementary Fig. 1b for gating strategy). A prominent change was observed within the macrophage population, where we observed a clear shift from proinflammatory M1 to the pro-regenerative M2 macrophage phenotype (defined as CD80^+^, MHCII^+^ and CD206^+^, respectively) (Fig. 2e and Supplementary Fig. 6b). Consistently, coculturing of purified and pre-activated NK cells (PMA/ionomycin) from WT and HIF-1α KO mice with splenocytes including monocytes and macrophages resulted in a tendency towards lower M1/M2 ratio in the HIF-1α null setting (Supplementary Fig. 6c). Moreover, NK cell depletion prior to wounding (as described in Fig. 1c) leads to a decrease of M1 macrophages at day 2 post-wounding (Supplementary Fig. 6d). The relative abundance of M2 macrophages in wounds from HIF-1α KO mice (Fig. 2e) likely contributes to increased angiogenesis as proangiogenic action is a hallmark of M2 macrophages^21^ (Fig. 1b). Macrophages can be further characterised by the expression of two competing enzymes. Inducible nitric oxide synthase (encoded by the gene *Nos2*) is predominantly expressed in the M1 subset whereas arginase 1 (encoded by the gene *Arg1*) is typically expressed by M2 macrophages. In addition, M2 macrophages express high levels of the cytokine *Il10*^1,21^. Wounds from HIF-1α KO mice exhibit a relative increase in the *Arg1*/*Nos2* expression ratio and increased *Il10* expression. (Fig. 2f, g). This is associated with a reduced expression of the proinflammatory cytokines Il1b and Il6 (Fig. 2g). Although, *Nos2* and *Arg1* can be expressed by various other cell types, these findings support the predominance of M2 macrophages upon loss of HIF-1α in NK cells.

The pronounced changes observed in wound healing provoked the question whether the deletion of HIF-1α in wound-infiltrating NK cells translates into global alterations of the early wound healing programme at a skin-wide level. We, thus, performed RNA sequencing on wounds from WT and HIF-1α KO mice at day 2 post wounding. The absence of HIF-1α in NK cells significantly changed the transcriptional programme and leads to differential gene expression with 304 up- and 282 downregulated genes (Fig. 2h). Gene ontology (GO) enrichment analysis associated the upregulated genes with positive Wnt signalling, fibroblasts growth factor signalling and cellular proliferation, hence, major repair response pathways (Fig. 2h and Supplementary Fig. 6e). The downregulated genes are involved in the coordination of leucocyte responses, including chemotaxis and cytokine signalling (Fig. 2h and Supplementary Fig. 6e). This indicates that deletion of HIF-1α in NK cells enhances a global pro-regenerative transcriptional programme in the skin while blunting pro-inflammatory responses. The expression of HIF-1α in NK cells accounts for systemic effects including macrophage polarisation, the regulation of wound angiogenesis and skin regeneration via early cytokine release.

### Constitutive HIF-1α expression in NK cells impairs skin regeneration

The profound effect of HIF-1α on wound closure prompted us to explore the impact of constitutive HIF activation in NK cells on skin regeneration. For this purpose, we created an NK cell-specific deletion of the negative HIF regulator VHL in NK cells, via crosses of the loxP-flanked *Vhl* allele^22^ to the *Ncr1* (NKp46) promoter-driven Cre recombinase^23^, (*Vhl*^fl+/fl+/^*Ncr1*^cre+^) termed VHL KO. First, we analyzed the skin from VHL KO mice and WT littermates at steady state. The skin from VHL KO mice contained similar frequencies and absolute numbers of NKp46+ cells, monocytes and macrophages, whereas neutrophils were modestly increased in VHL KO mice (Supplementary Fig. 7). Constitutive HIF activation in NKp46+ cells, thus, indeed promotes a low-grade proinflammatory response already at steady state conditions. It is important to note that the overall neutrophil numbers are very low compared to skin injury. This explains the absence of an obvious skin phenotype in VHL KO mice at steady state.

**Fig. 3:**
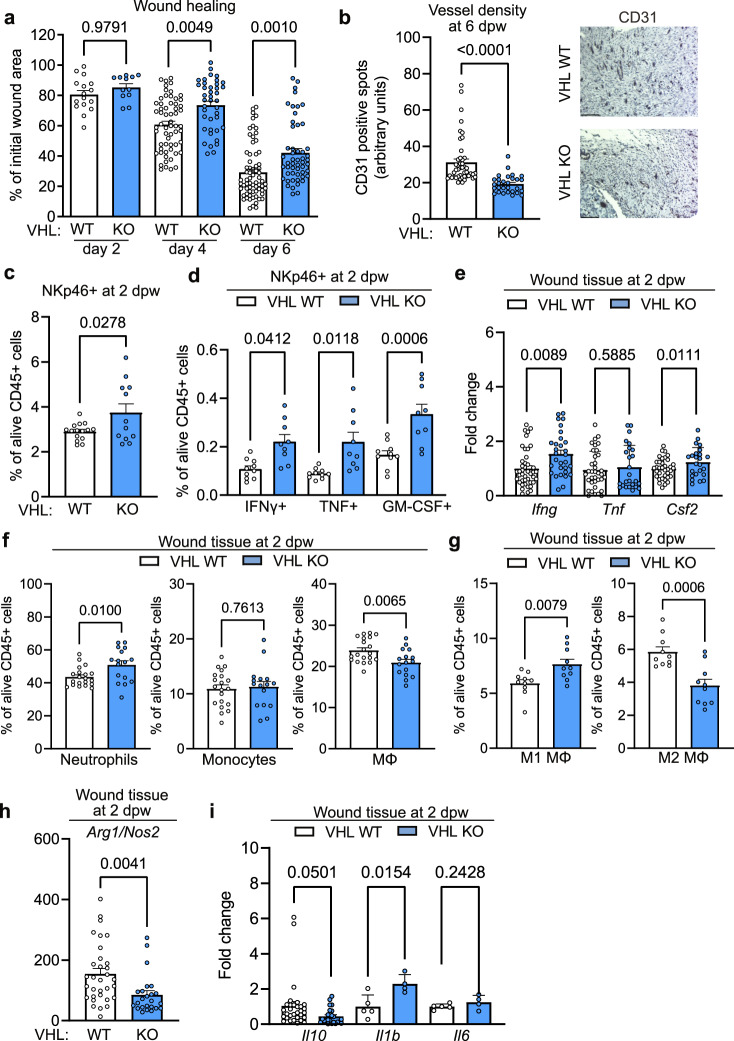
Constitutive expression of HIF-1α in NK cells impairs skin regeneration. **a** Wound areas at 2, 4 and 6 days post wounding (dpw), pooled data of 4 (day 4) and 5 experiments (day 6), (day 2, *n* = 16 for WT and *n* = 12 VHL KO; day 4, *n* = 60 for WT and *n* = 40 VHL KO; day 6, *n* = 68 for WT and *n* = 52 for VHL KO), one-way ANOVA test. **b** Left, quantification of CD31^+^ endothelial cells at 6 dpw (*n* = 46 for WT and *n* = 29 for VHL KO), pooled data of 3 experiments, two-tailed Student’s *t* test. Right, representative images of CD31 immunohistochemistry on skin wounds. Scale bar, 100 μm. FACS analysis at 2 dpw of (**c**) NKp46^+^ cells, pooled data of 3 experiments (*n* = 15 mice for WT and *n* = 12 for VHL KO), two-tailed Student’s *t* test, and (**d**) IFNγ-, TNF- and GM-CSF-expressing NKp46^+^, NK1.1^+^ cells, pooled data of 3 experiments (*n* = 10 mice for WT and *n* = 9 for VHL KO), two-tailed Student’s *t* test. **e** Gene expression of *Ifng, Tnf* and *Csf2* at 2 dpw, pooled data of 3 experiments (*Ifng*, *n* = 40 wounds for WT and *n* = 30 for VHL KO; *Tnf* *n* = 38 for WT and *n* = 30 for VHL KO; *Csf2*, *n* = 38 for WT and *n* = 30 for VHL KO), two-tailed Student’s *t* test. **f** FACS analysis at 2 dpw for neutrophils (CD11b^+^, Ly6G^+^), monocytes (CD11b^+^, Ly6C^+^), macrophages (CD11b^+^, Ly6C^+^, F4/80^+^), pooled data of 3 experiments (*n* = 20 mice for WT and *n* = 16 for VHL KO), two-tailed Student’s *t* test, and **g** M1 (CD80^+^, MHCII^+^) and M2 macrophages (CD206^+^), pooled data of 3 experiments (*n* = 10 mice), two-tailed Student’s *t* test. **h** Expression ratio for *Arg1* and *Nos2* at 2 dpw, pooled data of 3 experiments (*n* = 30 wounds for WT and *n* = 22 for VHL KO), two-tailed Student’s *t* test. **i** Expression of *Il10, Il1b* and *Il6* at 2 dpw, pooled data of 3 (*Il10*) and 2 experiments (*Il1b* and *Il6)*, (*Il10*, *n* = 30 wounds for WT and *n* = 20 for VHL KO, *n* = 4 for *Il1b* and *Il6*), two-tailed Student’s *t* test. Data are mean values ± SEM.

As VHL negatively regulates both HIF isoforms, the deletion of VHL not only stabilises HIF-1α but extends to the constitutive expression of HIF-2α. To control for these effects we crossed the loxP-flanked *Epas1* allele^24^ that encodes for HIF-2α to the *Ncr1* (NKp46) promoter-driven Cre recombinase^23^ (*Epas1*^fl+/fl+/^*Ncr1*^cre+^, termed HIF-2α KO). In this experimental setting we failed to detect any effects on the closure of punch biopsy wounds (Supplementary Fig. 8a), determining HIF-1α as the main mediator of hypoxic responses in wound-infiltrating NK cells. Constitutive HIF activation in NK cells slowed down wound closure (Fig. 3a) and reduced vascular density compared to WT littermates (Fig. 3b) in VHL KO mice thereby mirroring the effects seen in HIF-1α KO mice. Delayed wound healing was associated with a higher number of wound-associated NKp46^+^ cells (Fig. 3c and Supplementary Fig. 8b), and a discrete shift from NK cells to non-NK cell ILC1s in VHL KO mice (Supplementary Fig. 8c, d). NK cells remained the most abundant wound-infiltrating ILC1 subset in WT and VHL KO mice (≈90% and ≈80% of NKp46^+^, respectively) (Supplementary Fig. 8c, d). Moreover, we observed higher frequencies of IFN-γ^+^, GM-CSF^+^ and TNF^+^ NKp46^+^ cells in wounds derived from VHL KO mice (Fig. 3d) with significantly higher intracellular levels of IFN-γ and TNF, whereas GM-CSF levels were similar across genotypes (Supplementary Fig. 8e). The impact of NK cells was again confirmed by gene expression analysis of whole wounds and revealed increased levels of *Ifng* and *Csf2* but not *Tnf* (Fig. 3e). VHL loss in NK cells did not alter the frequency and number of wound-associated monocytes, but increased neutrophil counts and reduced macrophages count (Fig. 3f and Supplementary Fig. 8f). Moreover, this was associated with an increase of M1 and a reduction in M2 macrophages in frequency and absolute numbers (Fig. 3g and Supplementary Fig. 8g). Consistently, wounds from VHL KO mice exhibit a relative decrease in the *Arg1*/*Nos2* expression ratio as well as a decrease in IL 10 expression (Fig. 3h, i). This is associated with an elevated expression of the proinflammatory cytokine *Il1b* but not *Il6* (Fig. 3i). The relative abundance of M1 macrophages in wounds from VHL KO mice, hence, likely contributes to the reduced angiogenesis and to the impaired wound healing. This further corroborates the concept that NK cells orchestrate vascular remodeling and the immune response during skin regeneration via the cytokines IFN-γ and GM-CSF in a HIF-1α-dependent manner.

### HIF-1α in NK cells is required to control bacterial skin infections

**Fig. 4:**
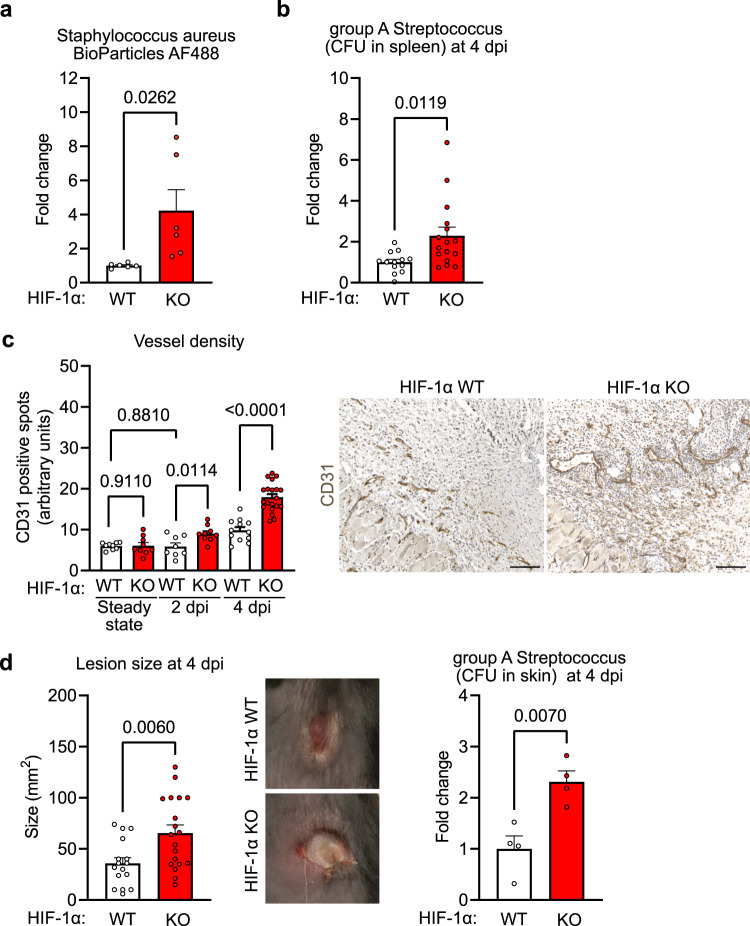
HIF-1α in NK cells is required to control bacterial skin infections. **a** Flow cytometry analysis of the spleen from WT and HIF-1α KO animals for Staphylococcus aureus bioparticles labelled with AlexaFluor 488 injected to the injury site, pooled of 3 experiments, two-tailed Student’s *t* test. **b** Colony forming units (CFU) of group A Streptococcus (GAS) in the spleen from WT and HIF-1α KO animals at 4 days post infection (dpi), pooled data of 4 experiments, (*n* = 13 for WT and *n* = 16 for HIF-1α KO), two-tailed Student’s *t* test. **c** Left, quantitative analysis of CD31-positive endothelial cells in skin lesions at 2- and 4- dpi with GAS from WT (steady state, *n* = 9; day 2, *n* = 8; day 4, *n* = 12) and HIF-1α KO animals (steady state, *n* = 9; day 2, *n* = 9; day 4, *n* = 20), pooled data of 3 experiments, one-way ANOVA test. Right, representative images of CD31 immunohistochemistry on skin wounds. Scale bar, 100 μm. **d** Left, quantification of skin lesion size at 4 dpi with GAS in WT and HIF-1α KO animals. Pooled data of 4 different experiments, (*n* = 16 lesions for WT and *n* = 19 for HIF-1α KO), two-tailed Student’s *t* test. Middle, macroscopic images of GAS skin lesions in WT and HIF-1α KO animals at 4 dpi. Right, GAS CFU in skin lesions from WT and HIF-1α KO animals at 4 dpi, (*n* = 4), two-tailed Student’s *t* test. Data are mean values ± SEM.

The observation that hypoxic NK cells decelerate skin remodelling after injury in a HIF-1α-dependent manner is intriguing as the process of skin regeneration in healthy individuals should already be optimised and take place at a maximum speed. Open wounds represent a potential infection site and rapid wound closure is thought to be essential to prevent the entry and subsequent dissemination of pathogens, predominantly bacteria^1,2^. One potential explanation for the evolutionary advantage of the delayed wound healing and reduced angiogenesis induced by hypoxic NK cells is the fact that vascular remodelling in wound margins eventually facilitates entry and dissemination of bacteria. We thus injected fluorescently labelled Staphylococcus aureus BioParticles into wound margins of WT and HIF-1α KO mice at day 4 post-wounding. Flow cytometric particle detection in the spleen was employed as a read-out for bacterial dissemination and revealed that the number of circulating particles in the spleen was increased by ≈4-fold in HIF-1α KO mice (Fig. 4a). This indicates facilitated bacterial translocation from the primary infection site to the blood in HIF-1α KO mice despite the more rapid wound healing. To support this conclusion, we made use of a well-established bacterial infection model by employing a strain of the Gram-positive pathogen group A *Streptococcus* (GAS). This strain has been isolated from a patient with necrotising fasciitis (flesh-eating disease)^11,12^. To test NK cell-mediated control of bacterial infections, we introduced the GAS inoculum subcutaneously into the shaved back skin of WT and HIF-1α KO littermates and followed progression of the infection over four days. GAS levels were significantly higher in cultures derived from the spleens of HIF-1α KO mice (Fig. 4b) paralleled by the increased vessel density in bacterial skin lesion (Fig. 4c) supporting the concept of facilitated bacterial dissemination across the vasculature. HIF-1α KO mice also developed significantly larger necrotic skin lesions with increased bacterial counts (Fig. 4d) suggesting enhanced uncontrolled local GAS replication. These findings verify the importance of HIF-1α in NK cells for limiting bacterial expansion at the initial focus of skin infection to prevent systemic spread.

### Hypoxic NK cells trigger an antibacterial defence programme

**Fig. 5:**
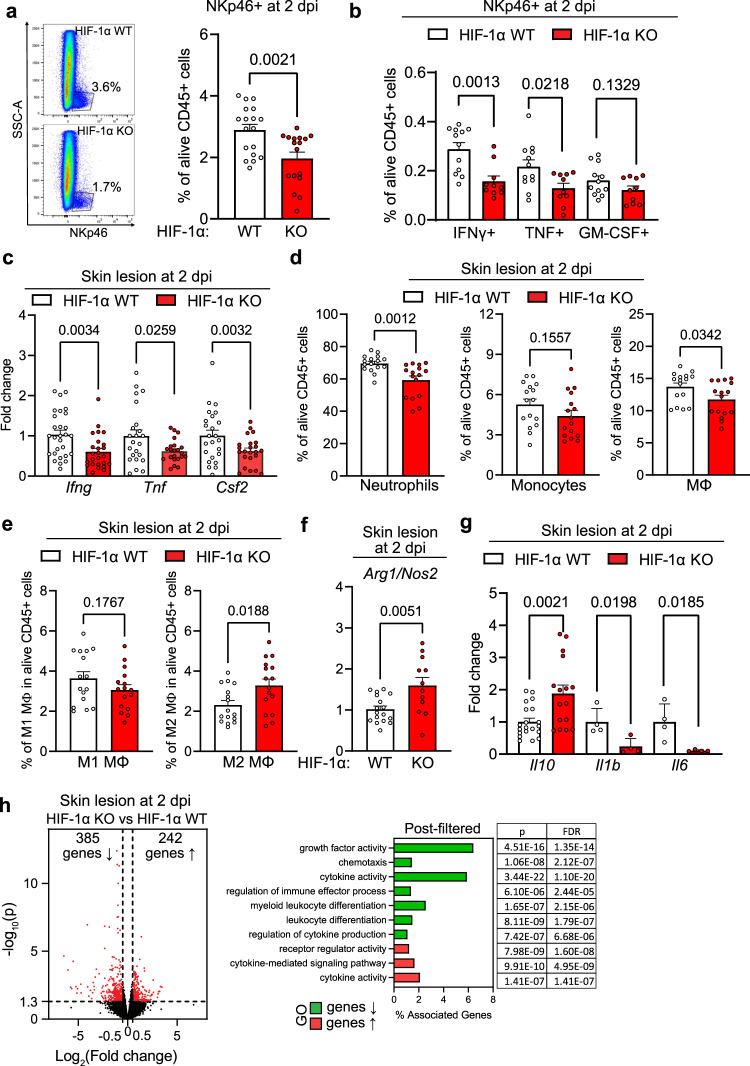
The hypoxic response in NK cells triggers an antibacterial defence programme. Analysis of GAS skin lesions from WT and HIF-1α KO mice at two days post infection (dpi). **a** Left, FACS plots showing the percentage of NKp46^+^ cells within CD45^+^ cells. Right, quantification of NKp46^+^ cells frequency, pooled data of 4 experiments (*n* = 18 mice for WT, *n* = 17 for HIF-1α KO). **b** FACS analysis of IFNγ-, TNF- and GM-CSF-expressing NKp46^+^, NK1.1^+^ cells in, pooled of 3 different experiments (*n* = 12 mice for WT and *n* = 10 HIF-1α KO). **c** Gene expression of *Ifng, Tnf* and *Csf2*, pool data of 4 experiments, (*Ifng*, *n* = 28 mice for WT, *n* = 22 for HIF-1α KO; *Tnf* *n* = 24 for WT, *n* = 24 for HIF-1α KO; *Csf2*, *n* = 24 for W, *n* = 24 for HIF-1α KO). **d** FACS analysis for neutrophils (CD11b^+^, Ly6G^+^), monocytes (CD11b^+^, Ly6C^+^) and macrophages (CD11b^+^, Ly6C^+^, F4/80^+^), pooled data of 4 experiments, (*n* = 16 mice). **e** FACS analysis for M1 (CD80^+^, MHCII^+^) and M2 macrophages (CD206^+^), pooled data of 4 experiments. (*n* = 16 mice). **f** Gene expression ratio for *Arg1* and Nos2, pooled data of 3 experiments. (*n* = 17 mice for WT, *n* = 12 for HIF-1α KO). **g** Gene expression for *Il10, Il1b* and *Il6*, pool data of 4 experiments for *Il10* and 2 experiments for *Il1b* and *Il6* (*Il10*, *n* = 18 mice WT, *n* = 16 for HIF-1α KO; *n* = 4 for *Il1b* and *Il6*). **h** Left, volcano plot for RNA sequencing analysis. Red data points meet the thresholds of log_2_(FC) above 0.5 and under −0.5, *P* < 0.05. Left, GO prediction of the post filtered genes for cytokines, chemokines and growth factors (including corresponding receptors) from the total pool of the significantly downregulated (385 genes) and upregulated genes (242 genes) with statistical readout (*p* value and FDR). Differential expression analysis was performed using the DESeq2 R package along with Wald test. Data are mean values ± SEM, statistical analysis: two-tailed Student’s *t* test.

In wound healing assays, loss of HIF-1α in NK cells had a profound impact as early as two days after injury. At that time point GAS skin lesions from HIF-1α KO mice presented with reduced percentages and absolute numbers of NKp46^+^ NK cells (Fig. 5a and Supplementary Fig. 9a) of which the vast majority (≈85−90%) were NK cells and only a minor fraction intILC1s (≈7−4%) and ILC1s (≈4–3%) (Supplementary Fig. 9b, c). Again, we observed a modest shift from intermediate ILC1s to NK cells in HIF-1α KO mice (Supplementary Fig. 9b). Similarly, the frequency of wound-infiltrating IFN-γ^+^, TNF^+^ and GM-CSF^+^ NKp46^+^ cells was reduced and HIF-1α null NKp46^+^ cells showed significantly lower levels of TNF and GM-CSF (Fig. 5b and Supplementary Fig. 9d). Gene expression analysis in whole skin lesions from HIF-1α KO mice revealed decreased expression of *Ifng, Tnf* and *Csf2* (Fig. 5c), along with a reduction in *sFlt1* expression (Supplementary Fig. 9e). Loss of HIF-1α in NK cells resulted in a decrease of neutrophils and macrophages, whereas the number of monocytes remained unchanged (Fig. 5d and Supplementary Fig. 9f), along with an increase in M2 macrophages in skin lesions (Fig. 5e and Supplementary Fig. 9f). Consistently, wounds from HIF-1α KO mice exhibit an increased *Arg1*/*Nos2* expression ratio (Fig. 5f), reduced expression of *Il1b* and *Il6* as well as a rise in *Il10* expression (Fig. 5g). Of note, neutrophils and M1 macrophages are crucial for the elimination of bacteria^1,12^, hence the relative paucity of the M1 subset in HIF-1α KO mice likely also contributes to the blunted antibacterial defence in these animals.

Finally, we asked to which extent the deletion of HIF-1α in NK cells alters the transcriptional programme in response to bacterial infections at a skin-wide level. RNA sequencing on bacterial skin lesions from WT and HIF-1α KO mice at day 2 post infection uncovered the downregulation of genes involved in the coordination of leucocyte responses, including chemotaxis and cytokine signalling (Fig. 5h and Supplementary Fig. 9h). This shows that deletion of HIF-1α in NK cells impairs the upregulation of an antimicrobial transcriptional programme at the whole skin level. In summary, this suggests that HIF-1α in NK cells is required to orchestrate vascular remodeling and the antimicrobial defence in the context of bacterial skin infections.

### Constitutive HIF-1α in NK cells improves the antibacterial defence

**Fig. 6:**
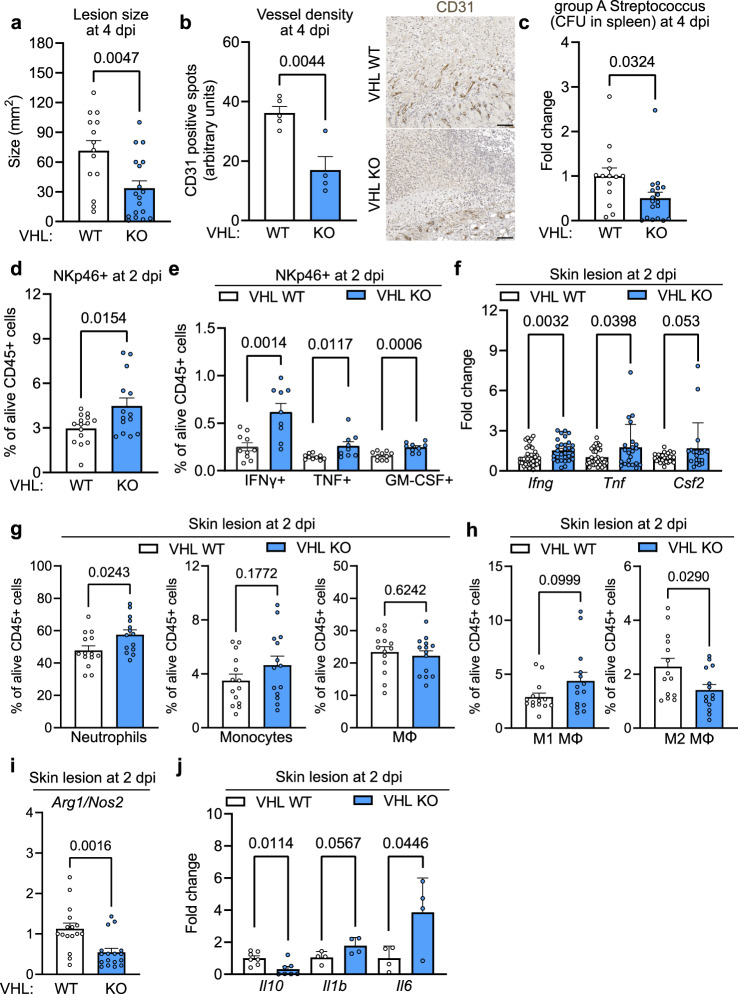
Constitutive HIF-1α expression in NK cells protects against bacterial skin infections. Analysis of GAS skin lesions from WT and VHL KO mice. **a** Quantification of independent skin lesion size area at 4 days post infection (dpi), pooled data of 4 experiments, (*n* = 14 mice for WT, *n* = 18 for VHL KO). **b** Left, quantification of CD31^+^ endothelial cells at 4 dpi, pooled data of 2 experiments (*n* = 5 for WT, *n* = 4 for VHL KO). Right, images of CD31 immunohistochemistry, scale bar, 100 μm. **c** CFU of splenic GAS at 4 dpi, pooled data of 4 experiments (*n* = 14 mice for WT and *n* = 18 for VHL KO). **d** FACS for NKp46^+^ cells in skin lesions at 2 dpi, pooled data of 4 experiments, (*n* = 15 mice for WT, *n* = 14 for VHL KO). **e** FACS analysis of IFNγ-, TNF- and GM-CSF-expressing NKp46^+^, NK1.1^+^ at 2 dpi, pooled data of 3 different experiments, (*n* = 10 mice for WT, *n* = 9 for VHL KO). **f** Gene expression of *Ifng, Tnf* and *Csf2* at 2 dpi, pooled data of 4 experiments (*Ifng*, *n* = 40 mice for WT, *n* = 30 for VHL KO; *Tnf* *n* = 40 for WT, *n* = 22 for VHL KO; *Csf2*, *n* = 30 for WT, *n* = 20 for VHL KO). **g** FACS analysis for neutrophils (CD11b^+^, Ly6G^+^), monocytes (CD11b^+^, Ly6C^+^) and macrophages (CD11b^+^, Ly6C^+^, F4/80^+^) at 2 dpi, pooled data of 4 experiments, (*n* = 14mice). **h** FACS for M1 (CD80^+^, MHCII^+^) and M2 macrophages (CD206^+^) at 2 dpi, pooled data of 4 experiments (*n* = 14 mice). **i** Expression ratio for *Arg1* and *Nos2* at 2 dpi, pooled data of 4 experiments, (*n* = 16 mice for WT, *n* = 16 for VHL KO). **j** Gene expression of *Il10, Il1b* and *Il6* at 2 dpi, pooled data of 3 experiments for *Il10* and of 2 experiments for *Il1b* and, (*n* = 7 mice for *Il10*; *n* = 4 for *Il1b* and *Il6*). Data are mean values ± SEM, statistical analysis: two-tailed Student’s *t* test.

GAS-infected VHL KO mice with constitutive HIF activation in NK cells developed significantly smaller necrotic skin lesions (Fig. 6a) with reduced vascular density (Fig. 6b) and decreased bacterial dissemination to the spleen (Fig. 6c). Thus, constitutive HIF activation protects against bacterial infections. GAS-infection in VHL KO mice induced higher frequency and numbers of NKp46^+^ NK cells (Fig. 6d and Supplementary Fig. 10a), along with a discrete shift from NK cells to intILC1s and ILC1s (Supplementary Fig. 10b, c). NK cells were the most abundant wound-infiltrating ILC1 subset in WT and VHL KO mice (≈82% and ≈70% of NKp46^+^, respectively) (Supplementary Fig. 10b). We observed higher frequencies of IFN-γ^+^, GM-CSF^+^ and TNF^+^ NKp46^+^ cells in wounds VHL KO mice (Fig. 6e) and VHL null NKp46^+^ cells also showed significantly increased intracellular levels of TNF, whereas IFN-γ and GM-CSF levels were similar across genotypes (Supplementary Fig. 10d). qPCR on whole lesions from VHL KO mice corroborated increased expression of *Ifng*, *Tnf* and *Csf2* (Fig. 6f), whereas *sFlt1* expression was similar across genotypes (Supplementary Fig. 10e). The loss of VHL in NK cells increased neutrophils (Fig. 6g and Supplementary Fig. 10f) and decreased M2 macrophages (Fig. 6h and Supplementary Fig. 10g), whereas the overall number of monocytes and macrophages remained unchanged (Fig. 6g and Supplementary Fig. 10f). This was associated with a reduced *Arg1*/*Nos2* expression ratio (Fig. 6i), increased expression of *Il1b*, *Il6* and decreased *Il10* expression in GAS lesions (Fig. 6j). The cytokine pattern as well as the relative abundance of M1 macrophages in lesions from VHL KO mice may contribute to reduced angiogenesis and improved control of bacterial infections.

In summary, our data suggest that HIF-1α in NK cells is critical for the antimicrobial defence in the skin.

### NK cells contribute to bacterial killing in a HIF-1α-dependent manner

**Fig. 7:**
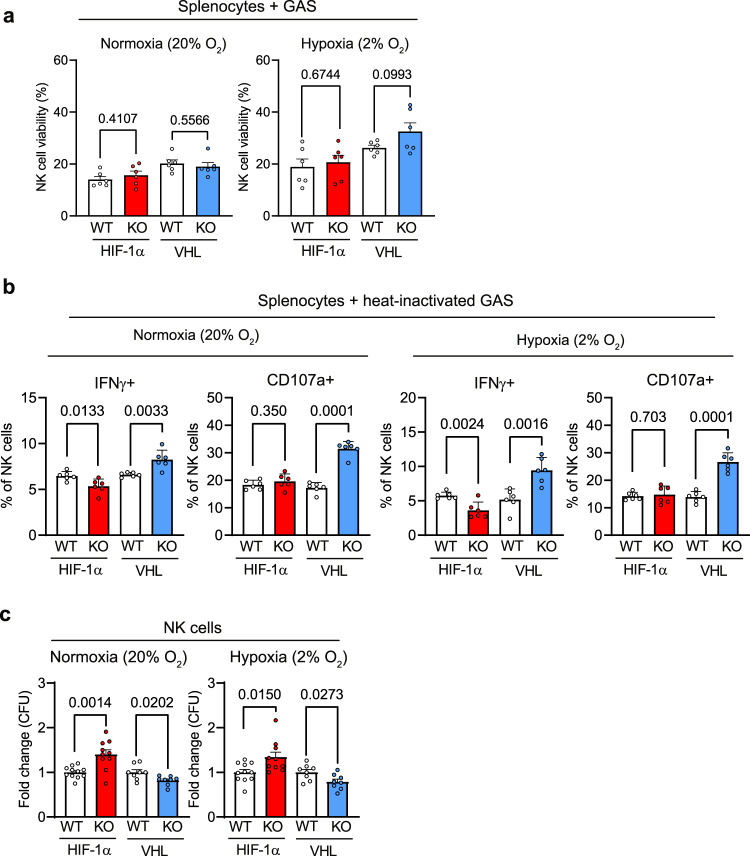
NK cells contribute to bacterial killing in a HIF-1α-dependent manner. **a** FACS analysis of NK cell viability after 4 h of coculture of splenocytes purified from HIF-1α WT, HIF-1α KO, VHL WT and VHL KO animals with GAS in normoxia (20% O_2_) or hypoxia (2% O_2_) relative to NK cell viability without GAS coculture, pooled data of 2, (*n* = 6 mice). **b** FACS analysis of NK cell (NKp46^+^, NK1.1^+^) activation (IFNγ^+^ and CD107a^+^) in splenocytes from HIF-1α WT, HIF-1α KO, VHL WT and VHL KO animals after stimulation with heat-inactivated GAS (hi GAS) for 6 h under normoxia (20% O_2_) and hypoxia (2% O_2_) relative to culture without GAS, pool data of 2 experiments, (*n* = 6). **c** Killing of GAS by purified splenic NK cells HIF-1α WT, HIF-1α KO, VHL WT and VHL KO. NK cells were cocultured with GAS at a multiplicity of infection (MOI) equal to 1 under normoxic (20% O_2_) or hypoxic (2% O_2_) conditions for 4 h. CFUs are represented as fold change relative to the corresponding WT NK cells, pooled data of 3 experiments, (*n* = 11 for HIF-1α WT, *n* = 10 HIF-1α KO, *n* = 8 for VHL WT and *n* = 8 for VHL KO). Data are mean values ± SEM, statistical analysis: two-tailed Student’s *t* test.

NK cells efficiently kill tumour cells^25^ and different microbial pathogens, including bacteria^7^. We, thus, questioned whether NK cells are activated by GAS and to which extend HIF-1α in NK cells modulates the direct elimination of bacteria. When we cocultured GAS with splenocytes from WT, HIF-1α KO and VHL KO mice in normoxia and hypoxia, NK cell survival was similarly reduced across genotypes (Fig. 7a). Bacteria can directly activate NK cells^7^, so we wanted to test, whether loss HIF-1α or VHL in NK cells affect NK cell activation upon exposure to GAS. As shown in Fig. 7b, upon challenge with heat-inactivated GAS, loss of HIF-1α decreases the number IFN-γ^+^ NK cells, whereas deletion of VHL increases the frequency IFN^+^ producing as well as CD107a^+^ degranulating NK cells.

It has been shown that NK cells can contribute to the elimination of bacteria by enhancing the bactericidal activity of myeloid cells as well as by direct killing of bacteria^7^. When we performed in vitro GAS killing assays, coculture of GAS with purified splenic NK cells from HIF-1α KO mice in 20% or 2% O_2_ resulted in increased CFU counts compared to WT controls (Fig. 7c), indicating that NK cell-mediated bacterial killing is impaired in the absence of HIF-1α. In contrast, purified NK cells from VHL KO mice lead to increased bacterial elimination as determined by reduced CFUs after coculture, in normoxia and hypoxia (Fig. 7c). Although the underlying mechanism remains to be elucidated, this strongly suggests that NK cells can contribute to the control of bacterial infections in a HIF-1α-dependent manner, and this also involves direct killing of bacteria.

Taken together, we show that in the absence of HIF-1α, NK cells drive an accelerated skin regeneration programme at the expense of increased susceptibility to bacterial infections and sepsis. In contrast, forced HIF-1α expression in NK cells boosts the antimicrobial defence in the skin at the expense of a limited wound healing pace. Therefore, our data suggest that HIF-1α in NK cells is crucial to balance antimicrobial defence versus repair mechanisms in the skin.

## Discussion

Here we report that mice lacking the HIF-1α isoform in NKp46^+^ cells show accelerated angiogenesis and repair but decreased bactericidal activity in the skin. This was associated with a reduced number of wound-infiltrating NKp46^+^ cells and impaired release of IFN-γ, GM-CSF and TNF in the absence HIF-1α.

One study showed that deletion of HIF-1α in NK cells isolated from MHC-1 deficient tumours at endpoint exhibited elevated effector function and IFN-γ release, with no impact on the tumour vasculature^18^. It is likely that this study describes the consequences of HIF-1α deletion in the context of a long-term tumour model (four weeks) of MHC-1 deficient tumours, whereas we demonstrate the impact of HIF-1α on acute hypoxia and the early NK cell cytokine response in context of skin repair and bacterial infection. Moreover, we noticed a decrease of NKp46^+^ cells in frequency and absolute number in the steady state skin of HIF-1α KO mice along with a proliferation defect in ex vivo-expanded HIF-1α KO NK cells. It is, hence, conceivable that loss of HIF-1α impairs local expansion of NKp46^+^ cells in the skin. This is in line with our previous report on tumour-infiltrating NKp46^+^ cells where HIF-1α-deficiency leads to a lower number of tumour-infiltrating NK cells with reduced effector function as well as lower levels of NK cell-derived antiangiogenic sFlt1, increased VEGF bioavailability and excessive angiogenic signalling in the tumour microenvironment^17^. Consistently, we observed reduced *sFlt1* expression in wound margins and GAS lesions from HIF-1α KO mice. In contrast to GM-CSF, which has been reported to support wound angiogenesis^26^, the NK cell-derived cytokine IFN-γ exerts a strong, direct antiangiogenic function on endothelial cells^10,27^, thus reduced IFN-γ levels in wounds and GAS lesions likely contribute to enhanced vascular remodelling in HIF-1α KO mice. In addition, IFN-γ and GM-CSF cooperate in the coordination of the early innate immune response and particularly M1 macrophage polarisation^1,21,28^. Consistent with reduced IFN-γ and GM-CSF levels in wound beds as well as GAS lesions from HIF-1α KO mice, we observe increased M2/M1 macrophage ratios. Of note, a hallmark of M2 macrophages is their proangiogenic function^1,21^ and the relative abundance of M2 macrophages is a likely contributor to enhanced angiogenesis in wounds and bacterial lesions of HIF-1α KO mice. In summary, we define robust antiangiogenic properties of hypoxic NK cells in the context of skin injury and infection. This is in contrast to a report showing that NKG2D receptor ligation improves wound healing^29^. However, the NKGD2 receptor is expressed on certain ILC subsets including NK cells as well as on CD8^+^ T cells and the effect on angiogenesis was not assessed^29^. Therefore, NKGD2 activation could facilitate wound closure via different cell types and independent of angiogenesis. Intriguingly, we demonstrate that ligation of NK1.1 receptor slows down angiogenesis in vitro in a HIF-1α-dependent manner. Therefore, it is conceivable that NK cells switch between pro- and anti-angiogenic properties in a stimulus- and receptor-specific manner. It is also a paradigm that uterine NK cells contribute to vascular remodelling during pregnancy^30,31^. However, the pO2 in the pregnant uterus^31,32^ does not reach the level of severe hypoxia in injured skin^1,2,11,12^. Moreover, the dynamic uterine vasculature also requires pruning of blood vessels^31^. Hence, NK cell-derived cytokines could participate in the coordinated expansion and regression of vasculature in a HIF-1α-dependent manner at distinct stages of pregnancy.

We report a pivotal role for NK cells for the antimicrobial defence in the skin. Noteworthy, non-NK cell ILC1s are known to play an important role in bacterial infections^28^ and NK cell-to-ILC1 conversion has been recently reported in malignant tumours^20^. We observe a discrete shift away from non-NK cell ILC1s towards NK cells in wounds and GAS lesions from HIF-1α KO mice. Therefore, hypoxia could contribute to the expansion of non-NK cell ILC1s in a HIF-1α-dependent manner. Yet, whether HIF-1α is required for NK cell-to-ILC1 conversion or local proliferation of skin-resident ILC1s, remains to be elucidated. However, the majority of NKp46^+^ ILC1s present in the skin upon wound healing and bacterial infection are NK cells and a recent study suggests that hypoxia does not contribute to NK cell-to-ILC1 conversion in malignant tumours^18^.

Hypoxia is a critical microenvironmental feature of bacterial skin infections and there is evidence that myeloid cells as well keratinocytes require HIF-1α to fight bacterial skin infections^11,12^. We now show that NK cells contribute to the early antibacterial defence at multiple levels and in a HIF-1α-dependent manner. The NK cell-derived cytokines IFN-γ and GM-CSF control vascular remodelling and transvascular dissemination of bacteria as well as the myeloid cell response upon GAS infection. Interestingly, we show direct killing of GAS by NK cells in a HIF-1α-dependent manner and this is in line with a previous report on impaired tumour cell killing by HIF-1α-deficient NK cells^17^. Although, the important role of myeloid cells for the elimination of bacteria is undisputable, it is conceivable that NK cells and other ILCs critically contribute to bacterial killing at early stages of bacterial infection before macrophages arrive in large numbers at the initial infection site^7^. Indeed, we demonstrate that GAS can activate NK cells, which results in killing of GAS by NK cells in HIF-1α-dependent manner. Although, the precise mechanism remains elusive, HIF-1α seems to be critically involved in direct bacterial killing. As HIF-1α KO animals fail to control GAS infection, this suggests that HIF-1α in NK cells drives direct bacterial elimination by NK cells as well as an early NK cell cytokine response to ensure an adequate myeloid cell response to complete bacterial elimination. Therefore, it will be important to elucidate in future studies the distinct pathways that are involved in NK cell-mediated GAS killing. Moreover, it will be important to study the hypoxic response in NK cells during infections with gram positive versus gram negative bacteria, including clinically highly relevant infections with Staphylococcus aureus and Pseudomonas aeruginosa^7,33^.

When we compared how the hypoxic response in skin-infiltrating NK cells affects global transcriptional programs in the skin, we found overlapping patterns between wound healing and bacterial infection. In both settings, NK cell-specific deletion of HIF-1α resulted in an enhanced pro-regenerative response and a blunted pro-inflammatory response at the skin-wide level. This further supports the conclusion that HIF-1α in skin-infiltrating NK cells differentially orchestrates immune responses and microbial defence versus a skin repair programme at the skin-wide level. During skin injury under sterile conditions, the absence of HIF-1α in NK cells accelerates skin repair, yet, at the expense of a compromised antimicrobial defence upon bacterial infection.

The critical role of HIF-1α is further substantiated as NK cell-specific deletion of VHL and forced activation of the HIF pathway supported cytokine release and NK cell-mediated antibacterial defence, including direct killing of bacteria by NK cells and despite delayed wound closure. In addition to potential HIF-independent effects of a VHL deletion in NK cells^34,35^, the deletion of VHL also results in constitutive expression of HIF-2α. However, NK cell-specific deletion of HIF-2α did not affect wound closure.

Conceptually, this suggests that adequate antimicrobial defence in the skin is achieved at the expense of a limited repair capacity, whereas an acceleration of physiological wound healing comes with a lower guard against infections. Hence, our data show that the transcription factor HIF-1α in NK cells is a key element to balance antimicrobial defence versus repair mechanisms in the skin.

## Methods

### Mouse models

Targeted deletion of HIF-1α, VHL and HIF-2α in NKp46-expressing NK cells was achieved by crossing the loxP-flanked *Hif1a* allele^17^, the loxP-flanked *Vhl* allele^22^ or loxP-flanked *Epas1* allele^24^ to the *Ncr1* (NKp46) promoter-driven cre recombinase (termed HIF-1α KO mice, VHL KO mice or HIF-2α KO mice). Cre recombinase negative mice from following strains were used as HIF-1α WT mice or VHL WT mice. To mitigate the influence of strain variation, mice were kept in a >99% C57Bl/6 J background. Mouse experiments were performed with at least three mice per group and multiple experiments were combined to assess statistically significant differences as noted. Littermates of the same genotype were randomly assigned to experimental groups. Animals were used between 8 and 12 weeks of age. All animal experiments have been approved by the veterinary authorities of Canton of Zurich, Switzerland, (licences ZH 219/2017 and ZH 169/2018) and were performed in accordance with Swiss law on the care, welfare, and treatment of animals.

### Excisional skin wounds

Following a protocol approved by the Zurich cantonal veterinary office, general anaesthesia of the animals was induced by 4% Isoflurane in 70% O_2_ and subsequently maintained using 3% Isoflurane. The back skin was shaved, thoroughly cleaned, and disinfected prior the surgery using 70% EtOH solution. Preoperative analgesia was applied using a subcutaneous injection of Buprenorphine (0.1 mg kg^−1^). Four circular full-thickness excisional wounds of 6 mm of diameter were generated on the lower back skin of each animal, two on each side, 1 cm of the midline of the animal and roughly 2 cm apart from each other. Post-operative analgesia was performed by a 5-day treatment of Buprenorphine through the drinking water.

### Macroscopic wound size measurement

Wound area was determined every two days under isoflurane anaesthesia for each wound by caliper measures taken from day 0 to day 6 post-surgery. The animals were monitored daily and criteria for early termination were: persistent non-closure of wounds or presence of pus in the wounds, persistent inactivity, persistent low responsiveness to handling, persistent skin tenting and persistent hunching, trembling, rapid and laboured breathing and weight loss >15%. The area at different time points after wounding were then reported to the initial area at day 0 for each wound.

### Depletion of NK cells during wound healing

Excisional wounds were generated at day −1 on the lower back skin of WT and HIF-1α KO mice. Mice were injected i.p. with anti-NK1.1 monoclonal antibody PK136 (4 mg per kg body weight; kindly provided by Prof. Veronika Sexl from Vienna) at day −2, 0, 2 and 4. Control mice received i.p. injections of 100 μl of isotype control.

### Bacterial strains and media

GAS strain 5448 is an M1 serotype isolate from a patient with necrotising fasciitis and toxic shock syndrome. GAS was propagated in Todd-Hewitt broth (THB) (Sigma, T1438)^36^.

### Mouse model of GAS infection

Briefly, 100 μl of a mid-logarithmic growth phase (~10^7^ CFU) of GAS was mixed with an equal volume of sterile Cytodex beads (Sigma, C0646) and injected subcutaneously into a shaved area on the flank of 8- to 12-week-old male and female littermates. Mice were weighed daily and monitored for development of necrotic skin lesions. After 48 or 96 h, skin lesions size was measured under isoflurane anaesthesia with a caliper and then collected together with spleen. Next spleen and skin lesion were homogenised in 1X PBS. Serial dilutions of the mixture were plated on THB agar plates for enumeration of CFUs. The animals were monitored daily and criteria for early termination were: persistent scratching or piloerection, persistent inactivity, persistent low responsiveness to handling, persistent skin tenting and persistent hunching, trembling, rapid and laboured breathing, oily or greasy fur and weight loss >15%. The area at different time points after wounding were then reported to the initial area at day 0 for each wound.

### Harvesting of skin tissue

At the day of sample collection, animals were euthanized using CO2 and skin lesions were excised post-mortem. For steady state analysis, a large flap of the shaved skin was removed followed by weighing of the tissue sample. Wound tissue was resected by excising a round 6 × 6 mm skin flap, which represents the initial excision wound size and therefore includes the wound and the skin around wound margins. Gas lesions were excised by removing a skin flap of 15 × 15 mm that includes the lesion as well as the adjacent inflamed skin.

### Single cell preparation from skin wounds and GAS lesions

Tissues were placed in gentleMACS C Tubes (Miltenyi, 130-093237) with 5 ml of HBSS/HEPES (w/ Ca^2+^, w/ Mg^2+^, 10 mM HEPES). Next, tissues were cut into small pieces in size of 1–2 mm^2^. Then, HBSS/HEPES was supplemented with 2 U/ml of Dispase II (Roche, 04942078001), 0.55 WU/ml of Liberase TL (Roche, 5401119001) and 200 KU/ml of DNAseI (Roche, 04716728001) and digestion reaction was performed at 37 °C with continuous shaking for 1 h. At the end of incubation, C Tubes were attached upside down onto the sleeve of the gentleMACS Dissociator and run twice for programme D. Cell suspensions were filtered through 70 µm CellStrainer and cells were washed twice with HBSS (w/o Ca^2+^, w/o Mg^2+^) buffer containing 2% FBS.

### Splenocyte isolation and stimulation

Splenic lymphocytes were prepared and cultured in the presence of PMA and ionomycin (eBioscience Cell Stimulation Cocktail (plus protein transport inhibitors), 00-4975-93) for 6 h at 37 °C in normoxia culturing conditions. Surface and intracellular stainings for TNF, GM-CSF and IFN-γ were performed using Cytofix/Cytoperm (BD-Bioscience, 554714). Cell viability was measured using LIVE/DEAD^®^ Fixable Aqua Dead Cell Stain Kit (Thermo Fisher, L34957).

### Flow cytometry

Single-cell suspension of skin wounds and skin lesions were obtained and stained. Cell viability was measured using LIVE/DEAD Fixable Aqua Dead Cell Stain Kit (Thermo Fisher, L34957). The following mAbs from eBioscience or BD-Biosciences or BioLegend were used: anti-F4/80 (BM8; 123107; 123131), anti-CD11c (N418; 117310;117306), anti-CD64 (X54-5/7.1; 139307), anti-CD8 (53-6.7; 100730), anti-CD206 (C068C2; 141732), anti-CD19 (6D5; 115530; 152404), anti-MHCII (M5/114.15.2; 48-5321; 107616), anti-Ly6G (1A8; 746448; 127618), anti-Ly6C (HK1.4; 128035), anti-CD80 (16-10A1; 104707), anti-CD45 (30-F11; 564225; 103128), anti-CD4 (GK1.5; 565974; 564667), anti-B220 (RA3-6B2; 564662), anti-CD11b (M1/70; 564443; 101206), anti-NK1.1 (PK136; 553165; 563220; 564144), anti-Siglec-F (E50-2440; 562757), anti-TCRβ (H57-597; 109210; 109206), anti-TCRγδ (GL3; 118106), anti-NKp46 (29A1.4; 25-3351), anti-TER119/Erythroid cells (TER-119; 116206), anti-CD127 (A7R34; 135027), anti-CCR6 (140706; 747831), anti-CD49b (DX5; 741752), anti-CD49a (Ha31/8; 741976), anti-ICOS (C398.4 A; 15-9949-82) and relevant isotype controls.

For detection of TNF, GM-CSF and IFN-γ, in brief, cells were stimulated by PMA and ionomycin (eBioscience Cell Stimulation Cocktail (plus protein transport inhibitors), 00-4975-93) for 4 h. Then, cells were stained with surface antibodies and fixed according to the manufacturer’s protocol eBioscience Intracellular Fixation & Permeabilization Buffer Set (88-8824-00). Next, cells were stained with anti-IFN-γ (XMG1.2; 566151), anti-TNF (MP6-XT22; 506333), anti-GM-CSF (MP1-22E9; 554406).

The nuclear stainings for RORγt (Q31-378; 562682), T-bet (4b10; 644835), GATA3 (TWAJ; 12-9966-42), and Eomes (Dan11mag; 61-4875-82) were performed according to manufacturer’s protocol eBioscience Foxp3/Transcription Factor Staining Buffer Set (00-5523-00).

The absolute count of specific cell subsets was determined by weighing the tissue mass was before single cell preparation from skin wounds and lesions. Next, total count of purified cells were counted using Trypan Blue staining. Cells were then stained (as described in Flow Cytometry) and prepared prior acquisition in equal volume and equal volume of sample was acquired using the BD High Throughput Sampler combined with BD Symphony A5.

Flow cytometry was carried out on a BD FACSymphony™ Flow CytometerFlow jo. Data were analysed using FlowJo v10 (Treestar).

### NK cell purification

NK cells were purified using NK Cell Isolation Kit II (Miltenyi, 130-115-818) and LS Column (Miltenyi, 130-042-401), and a MidiMACS Separator (Miltenyi, 130-042-302).

### Proliferation assay

NK cells were isolated from splenocytes using Stem Cell Technologies NK isolation kit (19855) as described by manufacturer. Cells were washed twice with warm (37 °C) PBS to remove serum that affects staining. Then, cells were suspended in warm (37 °C) PBS at a concentration of 1 × 106 cells/ml and labelled with 5 mM of CFSE (ThermoFisher, C34554) at 37 °C for 15 min. Subsequently, cells were washed with warm (37 °C) PBS and then were suspended in fresh medium (RPMI, 10% FBS, pen/strep) supplemented with 20 ng/mL IL-2 (PeproTech, 212-12), seeded on 96-well plates (2 × 105 cells/well) and incubated for 120 h. After this time, cells were collected and stained with LIVE/DEAD™ Fixable Near-IR Dead Cell Stain (ThermoFisher, L34975) (20 min, at 37 °C). Immediately after this step, cells were analyzed with flow cytometer (BD Symphony; BD Biosciences) with all necessary controls, i.e., CFSE negative, CFSE positive day 0 control.

### Migration assay

Cell migration was measured using a 24 well-format Transwell migration chamber (pore size, 5 µm, Corning, 3421). NK cells were isolated from splenocytes using Stem Cell Technologies NK isolation kit (19855) as described by manufacturer. First, NK cells were suspended in fresh medium (RPMI, 10% FBS, pen/strep) supplemented with 20 ng/mL IL-2 (PeproTech, 212-12) and seeded on 96-well plates (1 × 10^6^ cells/well). After 24 h, cells were washed with PBS and suspended in migration buffer (RPMI, 0.2% FBS) at 1 × 10^6^ cells/mL. For the migration to CCL5 chemokine, the migration buffer supplemented with 100 ng/mL CCL5 (PeproTech, 500-P118) was added in the lower compartment and 100 µl of cell suspension was added into each insert. After 4 h at 37 °C, the sample from lower compartment was collected and stained for NK1.1, NKp46 and Live/Dead marker (as described in Flow Cytometry) together with input sample. Samples were then suspended in equal volume and equal volume of sample was acquired using the BD High Throughput Sampler combined with BD Symphony A5 and the number of alive NKp46+, NK1.1+ positive cells per sample was analyzed. Migration rate was determined as a (nb of cells from sample / nb of cells from input sample) x 100%.

### Bacterial dissemination assay

Staphylococcus aureus (Wood strain without protein A) BioParticles conjugated with Alexa Fluor 488 (Thermo Fisher, S23371) were reconstituted in tissue culture-grade PBS at 20 mg/mL. Prior experiment, the 50-fold dilution was prepared with the tissue culture-grade PBS. BioParticles in volume of 25 µL were injected into wound margins of WT and HIF-1α KO mice at day 4 post-wounding. Mice were sacrificed 45 min post injection and single-cell suspension from spleen was analyzed by flow cytometry. Each splenic single-cell suspension was prepared in equal volume and equal volume of sample was acquired using the BD High Throughput Sampler combined with BD Symphony A5, followed by the analysis of circulating particles per spleen.

### Gene expression by quantitative PCR

General procedure: The tissue samples were homogenised in RLT buffer (Quiagen). Total RNA was isolated with Qiagen RNA extraction kits (Qiagen, 74104) following the manufacturer’s instructions. For real-time PCR analysis, the isolated RNA was reverse transcribed (High-Capacity cDNA Reverse Transcription Kit; Applied Biosystems, 4368814). For PCR reactions, SYBR Green I Master MIX (LightCycler 480 SYBR Green I Master, Roche, 04887352001) was used. 10 ng cDNA was used as template and the relative amount of mRNA was quantified by real-time PCR (LightCycler 96, Roche Detection System). PCR conditions were as follows: 95 °C for 10 min followed by 45 cycles of 95 °C for 15 s and 60 °C for 1 min. Data were normalised to 16 S mRNA levels.

For primers used in this study please see Supplementary Table 2.

### Scratch assay

We grew confluent monolayers of murine endothelial cells (mECs)^37,38^ in 96-well plates. A scratch wound in the confluent monolayer was generated by using a sterile 10-μl pipette tip. Next, we incubated the cells in a 1/1 mix of EBM-2 supplemented with VEGF-A (25 ng/ml) (Lonza, LONCC-3156) and the indicated splenocyte supernatants at 37 °C, 5% CO2 for 24 h. Pictures of scratch wounds were taken after wounding (time 0 h) and every 8 h with an automated Nikon Eclipse Ti microscope system. Wound scratches were analysed and scratch closure calculated with Nikon NIS elements software.

### Bacterial killing assays

GAS cultures were grown to logarithmic phase in THB to OD_600_ = 1 × 10^8^ cfu/ml. Bacteria were added to 10^5^ purified splenic NK cells at MOI of 1 bacteria/cell. Incubations were performed in normoxic (20% O_2_) and hypoxic (2% O_2_) conditions for 4 h. At the end of the assay, serial dilutions of the bacteria/cell suspension were plated on THB agar plates for enumeration of CFUs.

### In-vitro activation with GAS

GAS cultures were grown to logarithmic phase in THB to OD_600_ = 1 × 10^8^ cfu/ml. Centrifuged at 5000 g for 10 min and resuspended in PBS to reach OD_600_ = 1 × 10^8^ cfu/ml. Then, GAS were heat inactivated by incubation at 80 °C for 10 min and placed on ice to cool down. Heat-inactivated GAS were added to 10^6^ splenocytes at MOI of 1 bacteria/cell. Incubations were performed in 10% FBS RPMI + GlutaMax medium supplemented with GolgiStop/Plug (ThermoFisher, 554724, 555029) and anti-CD107a (1D4B, 560648) in normoxic (20% O_2_) conditions for 6 h. Activation of NK cells was analysed by flow cytometry using surface and intracellular staining for CD107a and IFN-γ (XMG1, 554413).

### Histological analysis of murine skin samples

At the day of sample collection, animals were euthanized using CO_2_ and skin samples were excised post-mortem, fixed in a buffered 4% formaldehyde solution at 4 °C, overnight for samples to be embedded in paraffin. Paraffin blocks were processed into sections of 5 µm. For immunofluorescence, sections were deparaffinised and subjected to a heat antigen retrieval step with citrate buffer (Dako, S2369). Prior incubation with antibodies, sections were blocked at RT for 1 h in blocking buffer (5% Normal Donkey Serum in PBS and 0.05% Tween-20). Primary antibodies were applied in blocking buffer overnight at 4 °C and visualised using secondary antibodies in blocking buffer for 1 h at room temperature. Hoechst 33342 (Sigma, 14533) usually was used as nuclear counterstain at a 1 µg ml^−1^ working concentration. For IHC/DAB sections were processed like in Immunofluorescence method with few modifications. First, prior blocking with blocking buffer, endogenous biotin was blocked using Avidin/Biotin blocking kit (VectorLabs, SP-2001) and activity of endogenous peroxidase was blocked using 0.3% H_2_O_2._ Second, primary antibody was visualised using ABC kit and DAB substrate according manufactures protocol (VectorLabs, PK-6100). Immunofluorescence-stained and IHC/DAB -stained sections were imaged using either a DMI 6000B microscope (Leica) or an Axio Scan.Z1 slide scanner (Zeiss). Image analysis and quantifications were performed using ImageJ 1.49 (National Institutes of Health, USA) and ZEN 3.3 Blue edition 3.3 (Zeiss) imaging software.

### Primary antibodies for immmunohistochemistry and immunofluorescence

(a) rat anti-CD31 at 1:10 dilution (DIANOVA; DIA-310) (b) goat anti-NKp46 at 3 µg/mL dilution (R&D; AF2225). The fluorochrome-conjugated Alexa 488 (A11055) and DyLight 594 (SA5-10028) were used as secondary antibodies (1:200).

### Histological analysis of the vessel density

Vessel density was analysed on three sections per wound or lesion on photographs (10x magnification) taken by using a bright field microscope. The images were converted into JPEG and CD31 positive spots/vessels were counted manually per given area using ImageJ software. Next, the number of CD31 positive spots were divided by the respective area to obtain the average vessel density expressed as arbitrary units.

### RNAseq

The skin wounds or skin lesions from three HIF-1α WT and HIF-1α KO animals were directly placed into RNA lysis buffer, homogenised and processed for subsequent RNA isolation using RNeasy Mini Kit (Qiagen, 74104). RNAseq from total RNA was performed by Novogene Europe. Gene ontology network analysis was performed with ClueGO2.5.8/Cytoscape 3^39,40^. First, differential gene expression analysis was performed using a minimum fold change of 1.3 and a *p* value inferior to 0.05. Second, ClueGO 2.5.8/Cytoscape 3 was used and over-represented GO terms in the biological process category were identified. Benjamini–Hochberg correction was performed for multiple testing-controlled *P* values. Significantly enriched terms were functionally grouped and visualised. The highest significant term of the group was displayed as leading term.

### Statistical analysis

Data are represented as mean values ± SEM. Statistical analysis was performed with the Prism 8.3 software (GraphPad Software). Statistical significance was determined by an unpaired Student’s *t* test, one-way analysis of variance and two-ways analysis of variance, where appropriate.

### Reporting summary

Further information on research design is available in the Nature Research Reporting Summary linked to this article.

## Supplementary information

Supplementary Information

Reporting Summary

## Data Availability

The differentially expressed genes from RNAseq of wound healing study have been deposited in the ArrayExpress database under the accession number: E-MTAB-10699. The differentially expressed genes from RNAseq of group A Streptoccocus infection study have been deposited in the ArrayExpress database under the accession number: E-MTAB-10700. Source data are provided with this paper.

## Source data

Source Data
