# Supplementary material for: NK cells in hypoxic skin mediate a trade-off between wound healing and antibacterial defence
Source: Nat Commun. 2021 Aug 4;12:4700. doi: 10.1038/s41467-021-25065-w (PMC8338923; doi:10.1038/s41467-021-25065-w)
Supplement: Supplementary file 1 — Supplementary Information [file 41467_2021_25065_MOESM1_ESM.pdf]

Supplementary Information

Hypoxic NK cells mediate a trade-off between wound healing and antibacterial defence in the skin

Michal Sobecki[1,§], Ewelina Krzywinska[1, §], Shunmugam Nagarajan[1], Annette Audigé[1], Khanh Huỳnh[1], Julian Zacharjasz[1], Julien Debbache[1], Yann Kerdiles[2], Dagmar Gotthardt[3], Norihiko Takeda[4], Joachim Fandrey[5], Lukas Sommer[1], Veronika Sexl[3] and Christian Stockmann[1*]

Inventory of Supplemental Information

I. Supplementary Figures

Supplementary Fig.1
Supplementary Fig.2
Supplementary Fig.3
Supplementary Fig.4
Supplementary Fig. 5
Supplementary Fig. 6
Supplementary Fig. 7
Supplementary Fig. 8
Supplementary Fig. 9
Supplementary Fig. 10

II. Supplementary Tables

Supplementary Table 1
Supplementary Table 2

Suppl. Fig. 1

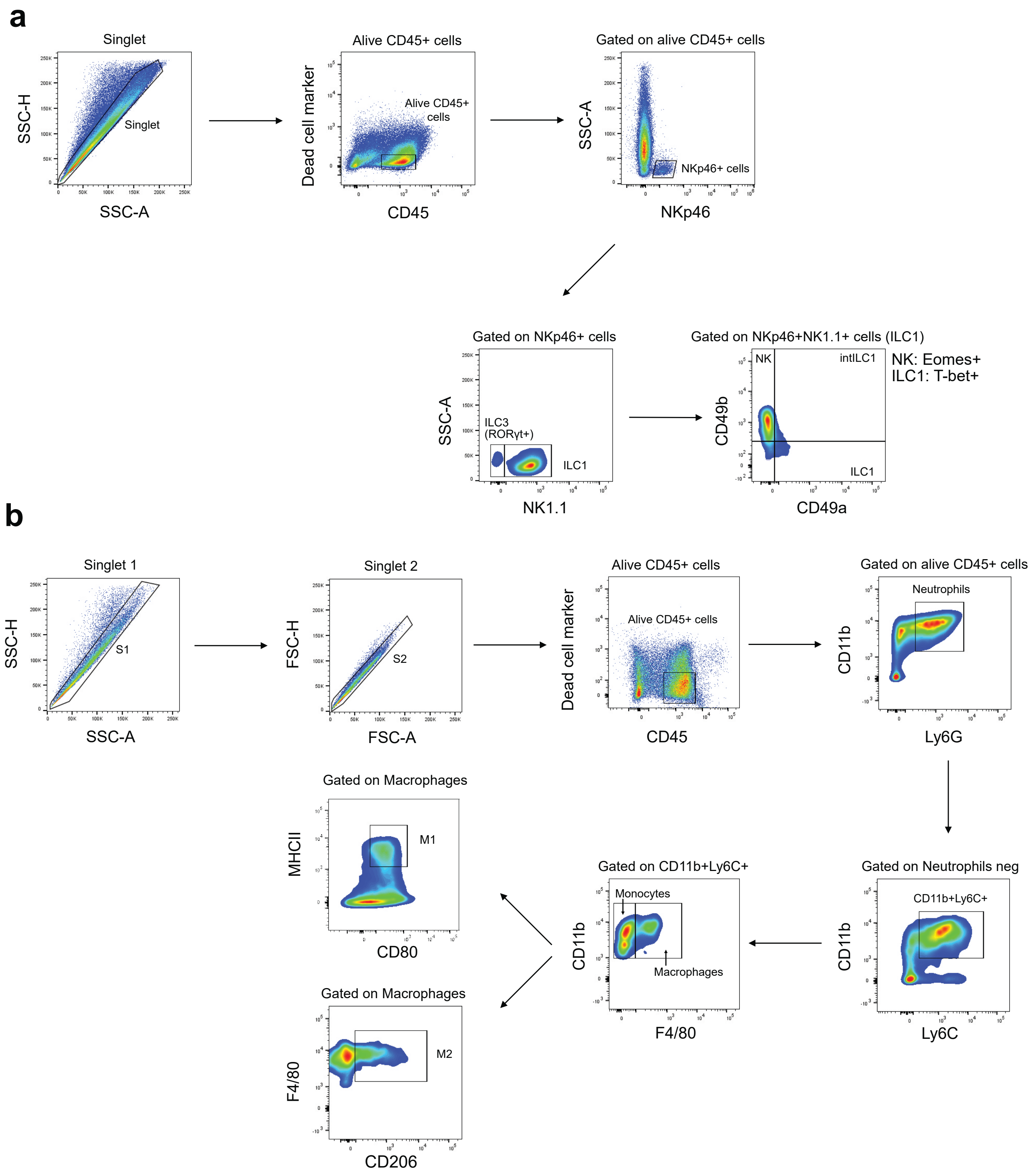

a
Singlet
SSC-H
SSC-A
Alive CD45+ cells
Dead cell marker
Alive CD45+ cells
CD45
Gated on alive CD45+ cells
SSC-A
NKp46+ cells
NKp46
Gated on NKp46+ cells
SSC-A
ILC3 (RORγt+)
ILC1
NK1.1
Gated on NKp46+NK1.1+ cells (ILC1)
NK
intILC1
ILC1
CD49b
CD49a
NK: Eomes+
ILC1: T-bet+
b
Singlet 1
S1
Singlet 2
FSC-H
S2
FSC-A
Alive CD45+ cells
Dead cell marker
Alive CD45+ cells
CD45
Gated on alive CD45+ cells
Neutrophils
CD11b
Ly6G
Gated on Neutrophils neg
CD11b+Ly6C+
Ly6C
Gated on CD11b+Ly6C+
Monocytes
Macrophages
F4/80
Gated on Macrophages
MHCII
M1
CD80
Gated on Macrophages
M2
F4/80
CD206

Supplementary figure 1. Gating strategy to detect immune cell subsets. a Gating strategy for NKp46$^+$, ILC1 group cells (NKp46$^+$, NK1.1$^+$), ILC3 cells (NKp46$^+$, NK1.1-, RORγt$^+$), NK cells (NKp46$^+$, NK1.1$^+$, CD49b$^+$, Eomes$^+$), intermediate ILC1 cells (NKp46$^+$, NK1.1$^+$, CD49b$^+$, CD49a$^+$) and ILC1 cells (NKp46$^+$, NK1.1$^+$, CD49a$^+$, CD49b$^-$, T-bet$^+$) cells population. **b** Gating strategy for neutrophils (CD11b$^+$, Ly6G$^+$), monocytes (CD11b$^+$, Ly6C$^+$), macrophages (CD11b$^+$, Ly6C$^+$, F4/80$^+$), M1 macrophages (CD80$^+$, MHCII$^+$) and M2 macrophages (CD206$^+$).

Suppl. Fig. 2

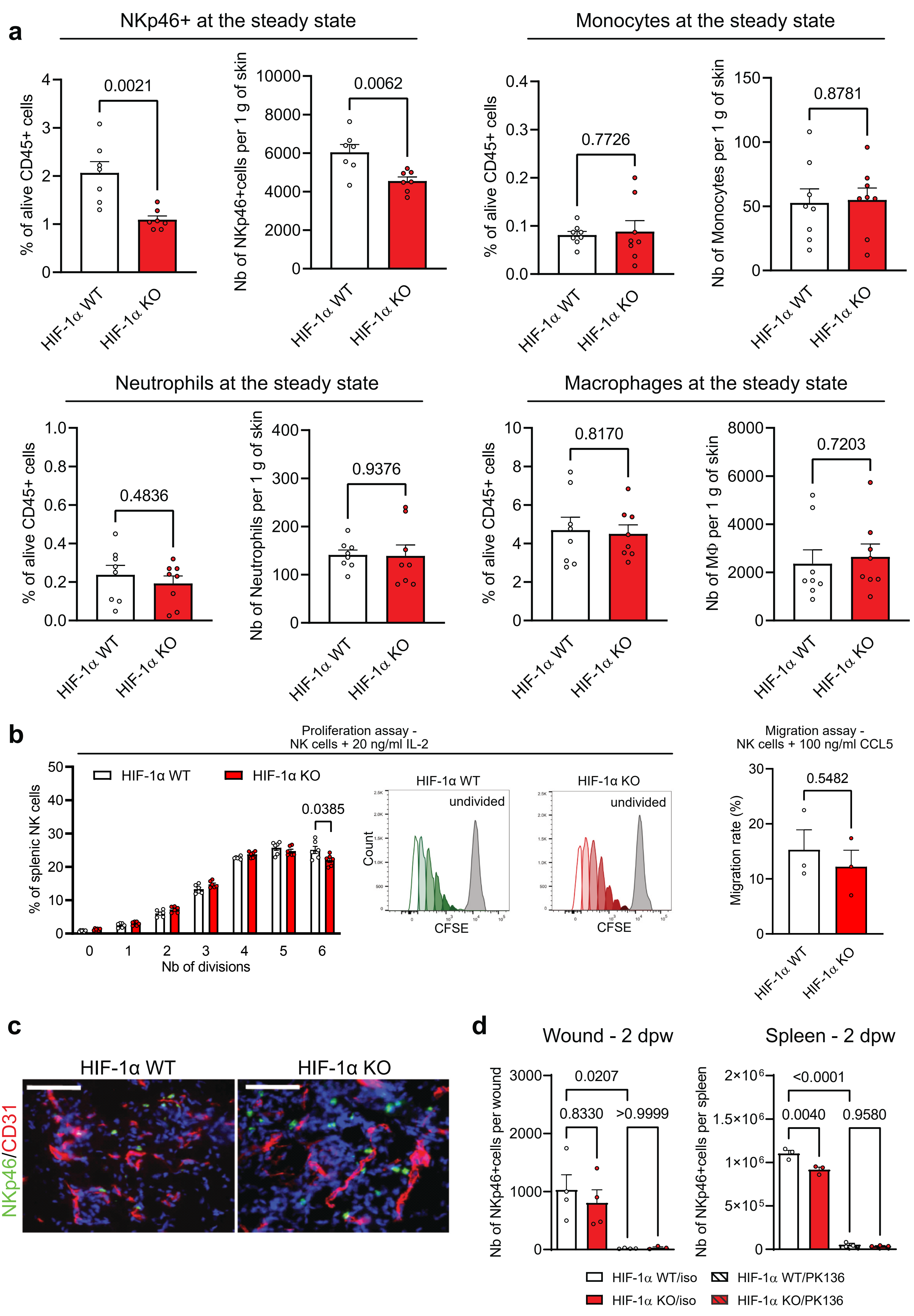
a
NKp46+ at the steady state
Monocytes at the steady state
% of alive CD45+ cells
Nb of NKp46+cells per 1 g of skin
Nb of Monocytes per 1 g of skin
0.0021
0.0062
0.7726
0.8781
HIF-1α WT
HIF-1α KO
Neutrophils at the steady state
Macrophages at the steady state
Nb of Neutrophils per 1 g of skin
Nb of MΦ per 1 g of skin
0.4836
0.9376
0.8170
0.7203
b
Proliferation assay -
NK cells + 20 ng/ml IL-2
% of splenic NK cells
Nb of divisions
0.0385
undivided
Count
CFSE
Migration assay -
NK cells + 100 ng/ml CCL5
Migration rate (%)
0.5482
c
NKp46/CD31
d
Wound - 2 dpw
Spleen - 2 dpw
Nb of NKp46+cells per wound
Nb of NKp46+cells per spleen
0.0207
0.8330
>0.9999
<0.0001
0.0040
0.9580
HIF-1α WT/iso
HIF-1α KO/iso
HIF-1α WT/PK136
HIF-1α KO/PK136

Supplementary figure 2. Loss of HIF-1α in NKp46$^+$ cells affects wound closure. a FACS analysis of NKp46$^+$ cells, neutrophils (CD11b$^+$, Ly6G$^+$), monocytes (CD11b$^+$, Ly6C$^+$) and macrophages (CD11b$^+$, Ly6C$^+$, F4/80$^+$), frequencies and absolute cell numbers per 1 gram of skin from WT and HIF-1α KO mice, pooled data of 2 experiments, (for NKp46$^+$ cells, n=7; for neutrophils, monocytes, macrophages, n=8), two-tailed Student's t test. **b** Left, analysis of purified splenic NK cells proliferation from HIF-1α WT and HIF-1α KO mice in the presence of IL-2 with corresponding FACS plots, (n= 6), two-tailed Student's t test. Right, analysis of purified splenic NK cell migration from WT and HIF-1α KO mice in the presence of CCL5 (n=3), two-tailed Student's t test. **c** Representative images of CD31/NKp46 immunostaining on skin wounds from WT and HIF-1α KO mice at day 6 post injury. **d** Left, flow cytometry analysis in skin wounds for NK cells (NKp46$^+$, NK1.1$^+$) absolute numbers from isotype control (iso) and PK136 antibody treated WT and HIF-1α KO animals at day 2 post injury. Right, flow cytometry analysis of splenic NK cells (NKp46$^+$, NK1.1$^+$) absolute numbers from isotype control- (iso) and PK136 antibody-treated WT and HIF-1α KO animals at day 2 post injury, (wounds n = 4; spleens, n=3), two-ways ANOVA test. Data are mean values ± SEM.

Supp. Fig. 3

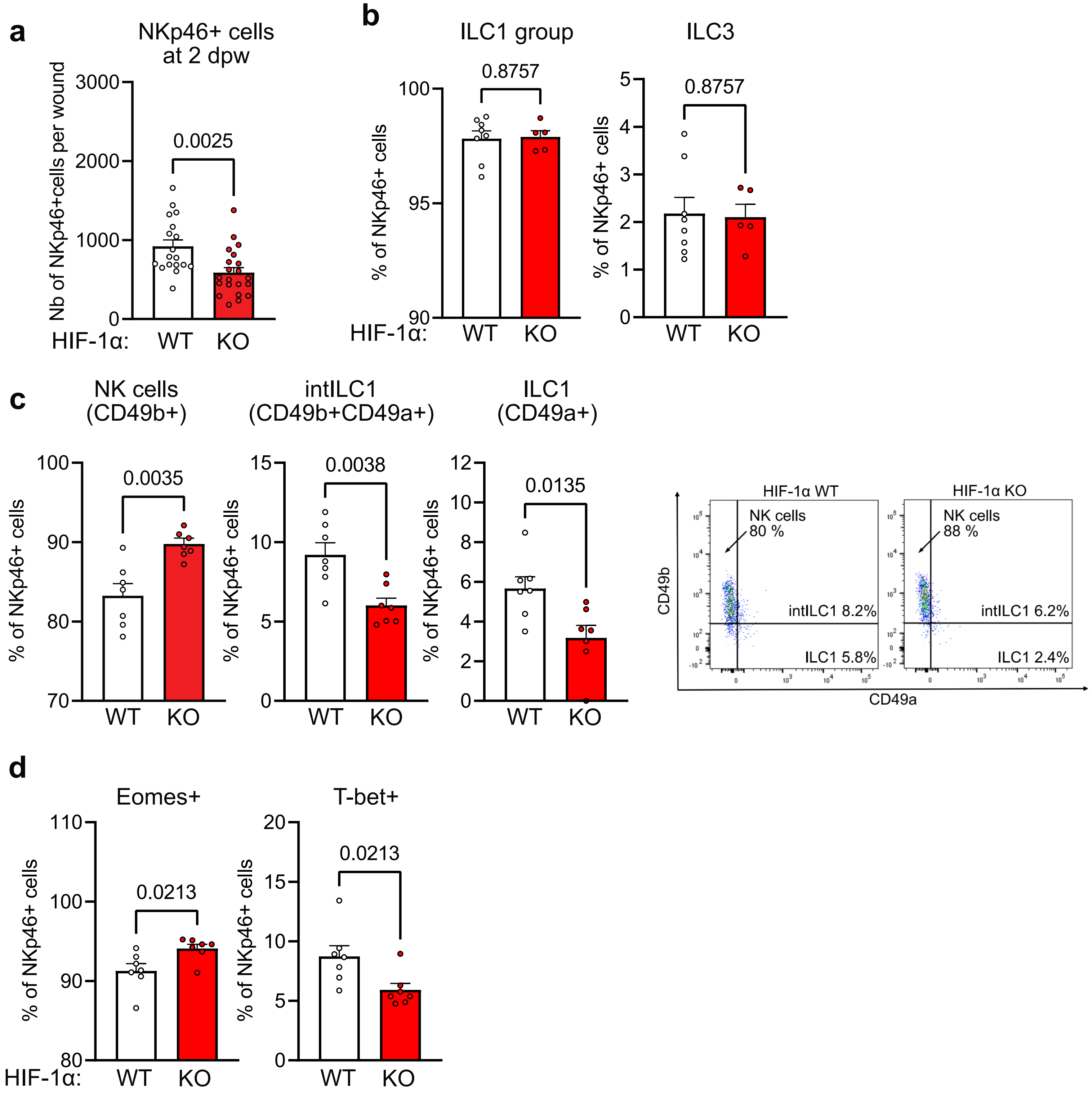
a
NKp46+ cells
at 2 dpw
Nb of NKp46+cells per wound
3000
2000
1000
0
0.0025
HIF-1α:
WT
KO
b
ILC1 group
ILC3
% of NKp46+ cells
100
95
90
0.8757
5
4
3
2
1
0
HIF-1α:
WT
KO
c
NK cells
(CD49b+)
intILC1
(CD49b+CD49a+)
ILC1
(CD49a+)
% of NKp46+ cells
100
90
80
70
0.0035
15
10
5
0
0.0038
12
10
8
6
4
2
0
0.0135
WT
KO
HIF-1α WT
HIF-1α KO
NK cells
80 %
88 %
intILC1 8.2%
ILC1 5.8%
intILC1 6.2%
ILC1 2.4%
CD49b
CD49a
d
Eomes+
T-bet+
% of NKp46+ cells
110
100
90
80
0.0213
20
15
10
5
0
HIF-1α:
WT
KO

Supplementary figure 3. The role of HIF-1α in NKp46^{+} cells for ILC composition in wounds. a Quantitative analysis by flow cytometry of NKp46^{+} cells, absolute numbers in skin wounds from WT and HIF-1α KO mice at day 2 post injury, pooled data of 4 experiments (n=18 for WT and n=21 for HIF-1α KO). **b** Flow cytometry analysis for ILC1 cells (NKp46^{+}, NK1.1^{+}), ILC3 cells (NKp46^{+}, NK1.1^{-}, RORγt^{+}) in skin wounds from WT and HIF-1α KO mice at day 2 post injury, pooled data of 2 experiments (n=8 for WT and n=5 for HIF-1α KO). **c** Left, flow cytometry analysis for NK cells (NKp46^{+}, CD49b^{+}), intermediate ILC1 cells (NKp46^{+}, CD49b^{+}, CD49a^{+}) and ILC1 cells (NKp46^{+}, CD49a^{+}, CD49b^{-}) in skin wounds from WT and HIF-1α KO mice at day 2 post injury, pooled data of 3 experiments (n=7). Right, corresponding representative FACS plots. **d** Flow cytometry analysis for NKp46^{+}, NK1.1^{+}, Eomes^{+} NK cells and NKp46^{+}, NK1.1^{+}, T-bet^{+} ILC1 cell populations in skin wounds from WT and HIF-1α KO mice at day 2 post injury, pooled data of 3 experiments (n=7). Data are mean values ± SEM, statistical analysis: two-tailed Student's t test.

Supp. Fig. 4

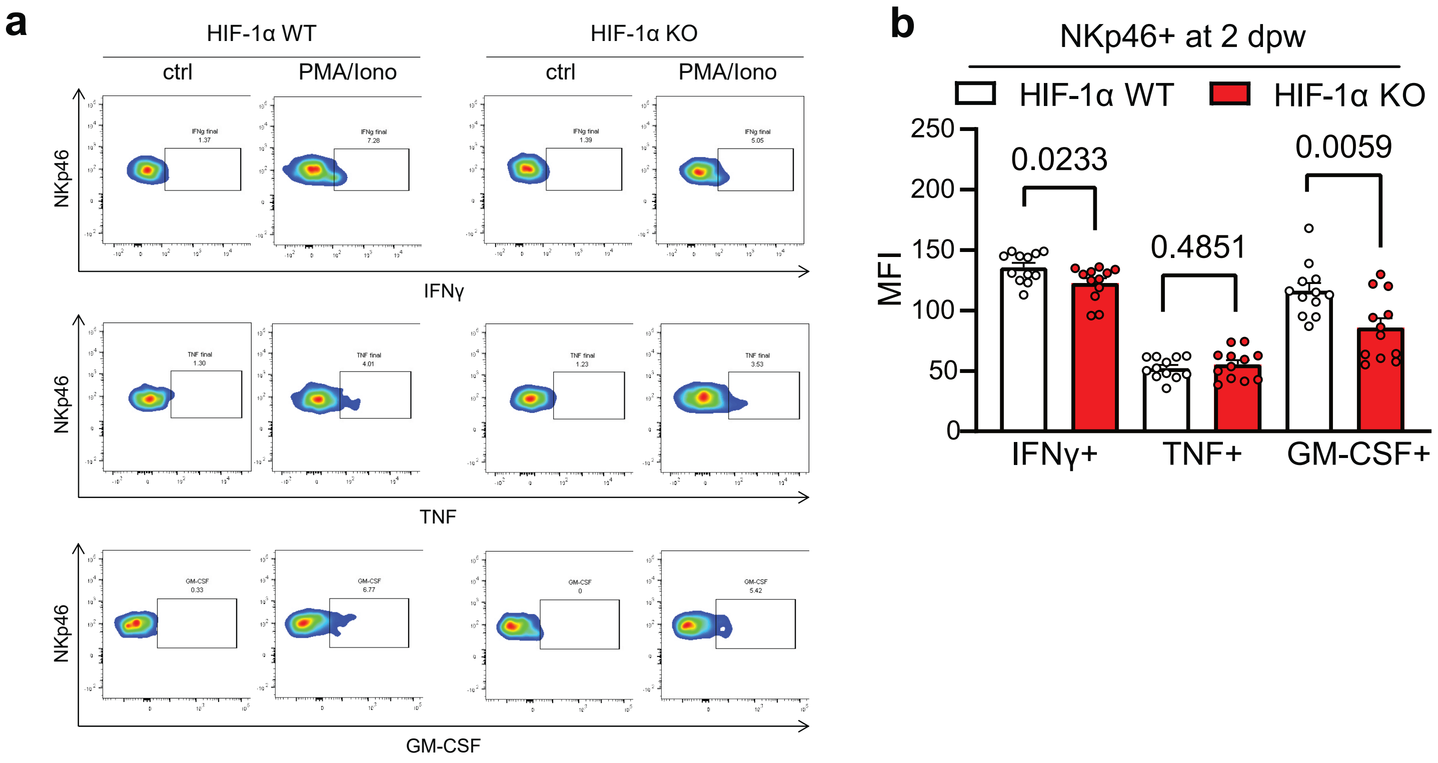
a
HIF-1α WT
HIF-1α KO
ctrl
PMA/Iono
NKp46
IFNγ
TNF
GM-CSF
b
NKp46+ at 2 dpw
HIF-1α WT
HIF-1α KO
MFI
0.0233
0.4851
0.0059
IFNγ+
TNF+
GM-CSF+

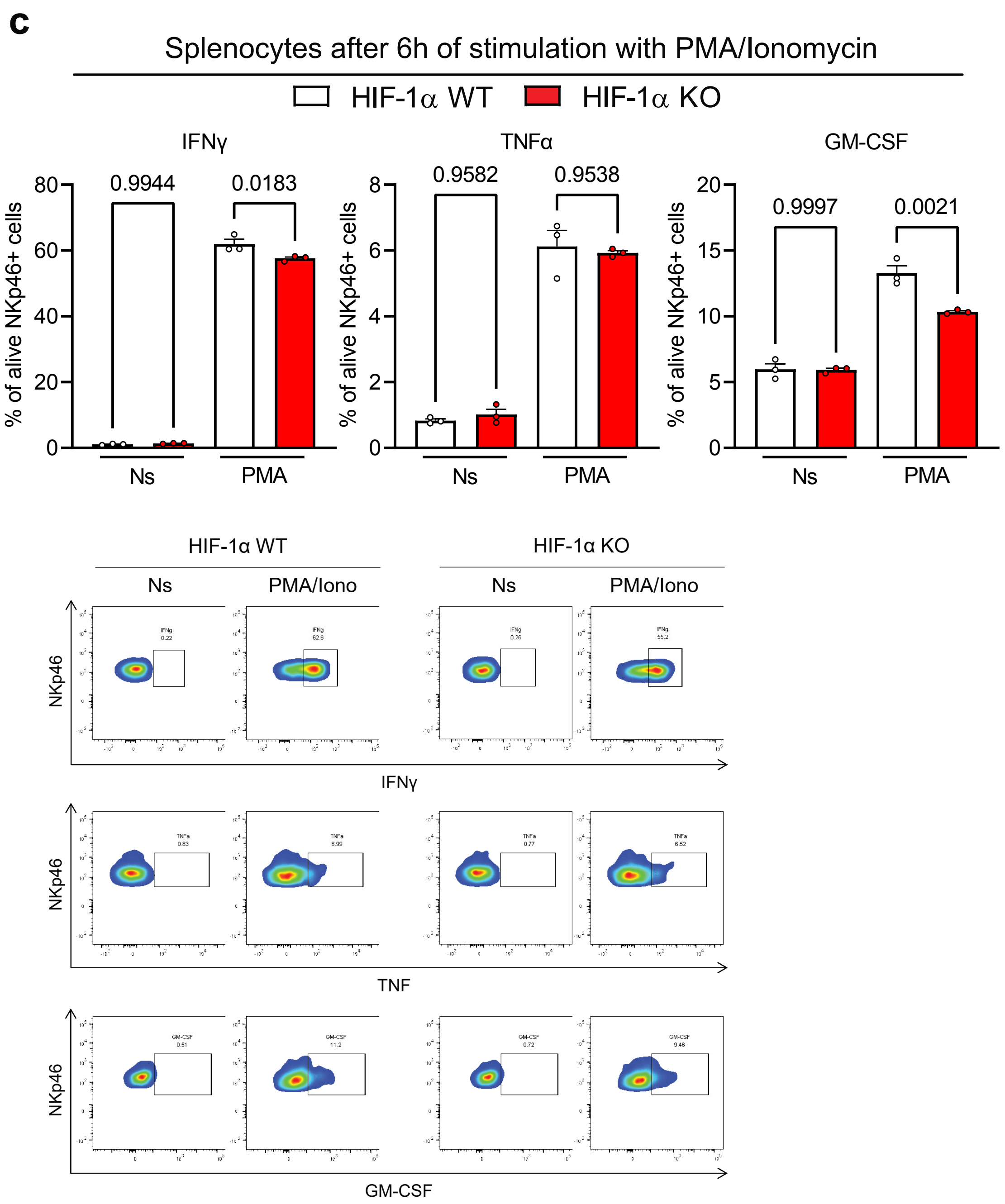
c
Splenocytes after 6h of stimulation with PMA/Ionomycin
HIF-1α WT
HIF-1α KO
IFNγ
TNFα
GM-CSF
% of alive NKp46+ cells
0.9944
0.0183
0.9582
0.9538
0.9997
0.0021
Ns
PMA
HIF-1α WT
HIF-1α KO
Ns
PMA/Iono
NKp46
IFNγ
TNF
GM-CSF

Supplementary figure 4. The effect of HIF-1α on NKp46$^+$ cell-derived cytokines. a Representative FACS plots showing the frequencies of IFNγ-, TNF- and GM-CSF-expressing NKp46$^+$, NK1.1$^+$ cells in skin wounds from WT and HIF-1α KO mice at day 2 post injury after stimulation with PMA/Inonomycin (Iono) and corresponding unstimulated controls (ctrl). **b** Flow cytometry analysis of mean fluorescence intensity (MFI) of IFNγ, TNFα and GM-CSF in NKp46$^+$, NK1.1$^+$ cells, pooled data of 3 experiments (n=12), two-tailed Student's t test. **c** Top, flow cytometry analysis of IFNγ-, TNF- and GM-CSF-expressing splenic NKp46$^+$, NK1.1$^+$ cells upon stimulation with PMA/Ionomycin together with corresponding controls. Values are mean ± SEM (n = 3) with significant differences by two-ways ANOVA test. Bottom, representative FACS plots. Data are mean values ± SEM.

Supp. Fig. 5

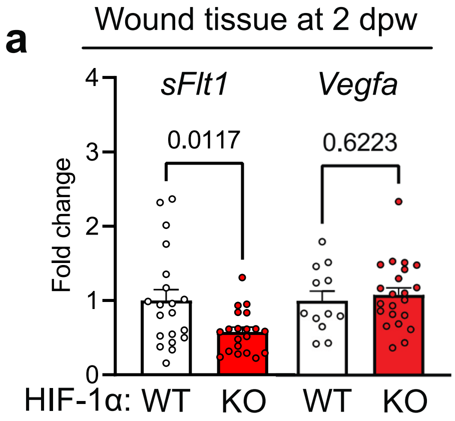
a
Wound tissue at 2 dpw
sFlt1
Vegfa
0.0117
0.6223
Fold change
0
1
2
3
4
HIF-1α: WT
KO
WT
KO

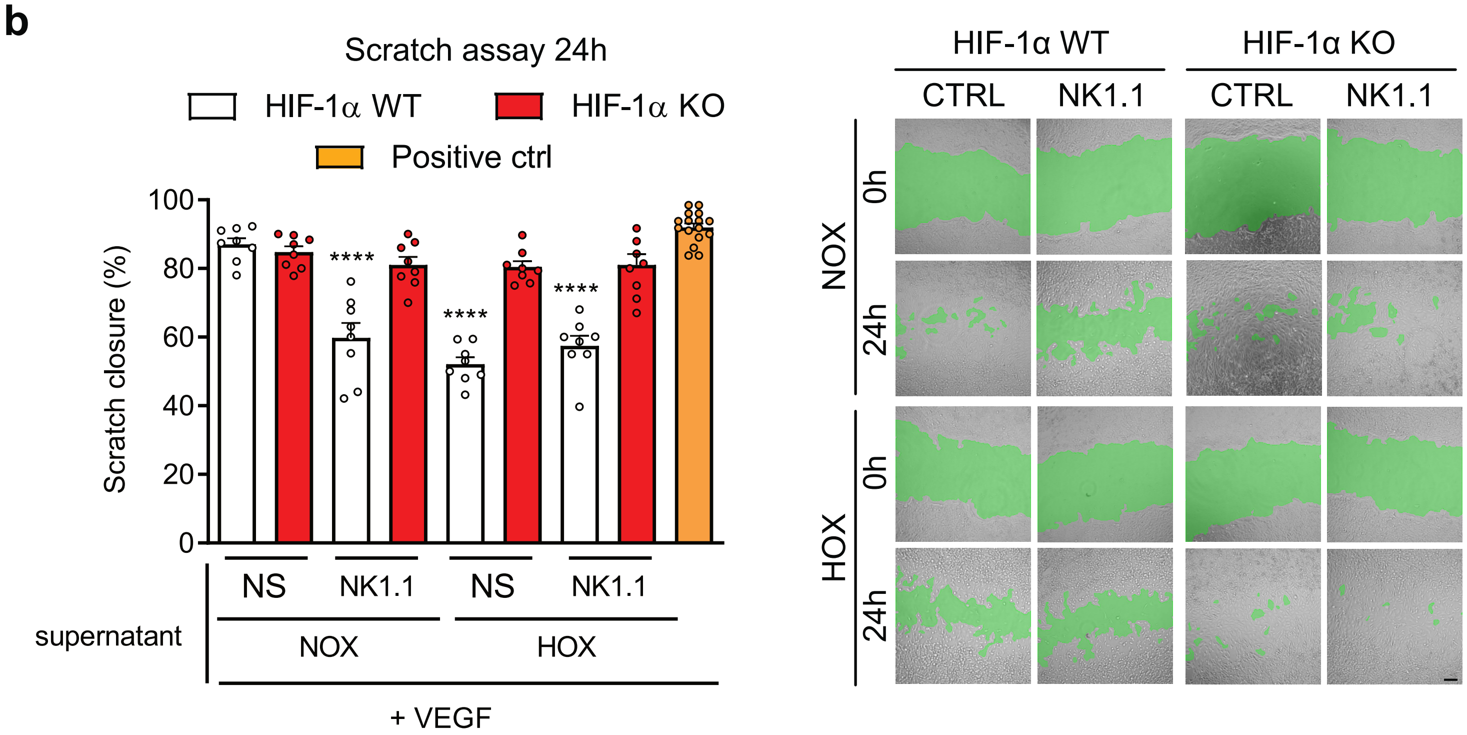
b
Scratch assay 24h
HIF-1α WT
HIF-1α KO
Positive ctrl
Scratch closure (%)
0
20
40
60
80
100
****
****
****
NS
NK1.1
NS
NK1.1
supernatant
NOX
HOX
+ VEGF
HIF-1α WT
HIF-1α KO
CTRL
NK1.1
CTRL
NK1.1
NOX
HOX
0h
24h
0h
24h

Supplementary figure 5. Loss of HIF-1α in NKp46^{+} cells facilitates angiogenesis. a Gene expression analysis of *sFflt1* (left) and *Vegfa* (right) in skin wounds of WT and HIF-1α KO animals at day 2 post injury (n=20), two-tailed Student's t test. **b** Left, endothelial cell scratch assay, quantification of scratch closure relative to the initial scratch size at time point 0h, (n = 8, for positive control n=16), two-ways ANOVA test. *** $P < 0.001$, and **** $P < 0.0001$. Right, representative images of the scratch assay with the scratch area highlighted in green. Data are mean values ± SEM.

Supp. Fig. 6

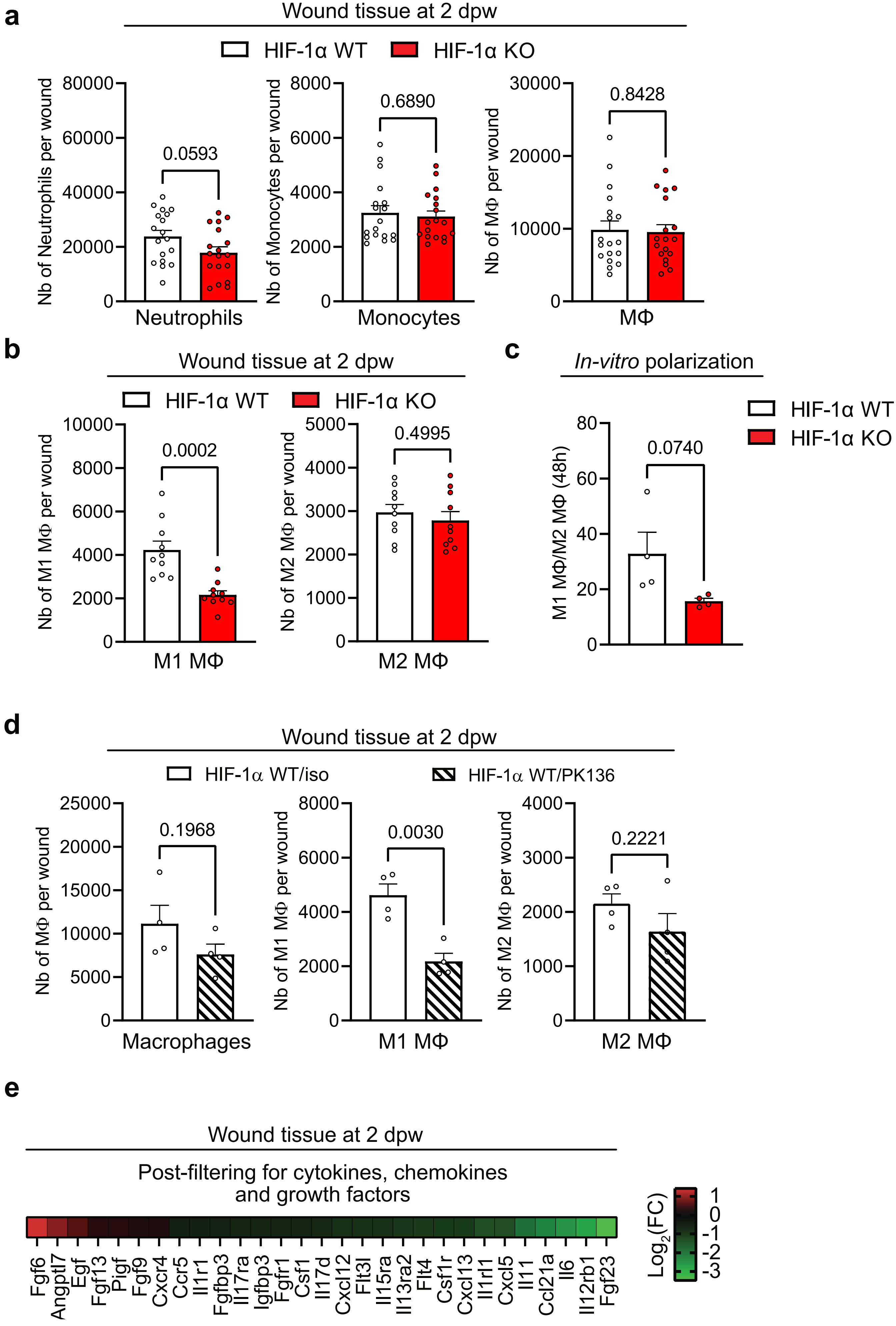

a
Wound tissue at 2 dpw
HIF-1α WT
HIF-1α KO
Nb of Neutrophils per wound
0.0593
Neutrophils
Nb of Monocytes per wound
0.6890
Monocytes
Nb of MΦ per wound
0.8428
MΦ
b
Wound tissue at 2 dpw
HIF-1α WT
HIF-1α KO
Nb of M1 MΦ per wound
0.0002
M1 MΦ
Nb of M2 MΦ per wound
0.4995
M2 MΦ
c
In-vitro polarization
HIF-1α WT
HIF-1α KO
M1 MΦ/M2 MΦ (48h)
0.0740
d
Wound tissue at 2 dpw
HIF-1α WT/iso
HIF-1α WT/PK136
Nb of MΦ per wound
0.1968
Macrophages
Nb of M1 MΦ per wound
0.0030
M1 MΦ
Nb of M2 MΦ per wound
0.2221
M2 MΦ
e
Wound tissue at 2 dpw
Post-filtering for cytokines, chemokines and growth factors
Fgf6 Angptl7 Egf Fgf13 Pigf Fgf9 Cxcr4 Ccr5 Il1r1 Fgfbp3 Il17ra Igfbp3 Fgfr1 Csf1 Il17d Cxcl12 Flt3l Il15ra Il13ra2 Flt4 Csf1r Cxcl13 Il1rl1 Cxcl5 Il11 Ccl21a Il6 Il12rb1 Fgf23
Log2(FC)

Supplementary figure 6. The role of HIF-1α in NKp46$^+$ cells for myeloid cell composition in wounds. a Flow cytometry analysis for neutrophils (CD11b$^+$, Ly6G$^+$), monocytes (CD11b$^+$, Ly6C$^+$) and macrophages (CD11b$^+$, Ly6C$^+$, F4/80$^+$), absolute cell numbers in skin wounds from WT and HIF-1α KO mice at day 2 post injury, pooled data of 4 experiments (n=18). **b** Flow cytometry analysis for M1 (CD80$^+$, MHCII$^+$) and M2 macrophages (CD206$^+$), absolute numbers in skin wounds from WT and HIF-1α KO mice at day 2 post injury, pooled data of 3 experiments, (n=10). **c** Flow cytometry analysis for M1 (CD80$^+$, MHCII$^+$) and M2 macrophages (CD206$^+$) derived from splenocytes from WT and HIF1a KO mice cocultured with pre-activated NK cells (PMA/ionomycin for 6 hours in normoxia) for 48 h (n=4). **d** Flow cytometry analysis in skin wounds for total macrophages (CD11b$^+$, Ly6C$^+$, F4/80$^+$), M1 macrophages (CD80$^+$, MHCII$^+$) and M2 macrophages (CD206$^+$), absolute numbers from isotype control (iso) and PK136 antibody treated HIF-1α WT animals at day 2 post injury (n = 4). **e** Heat map of gene expression in skin wounds of HIF-1α KO mice with a significantly differential expression to the skin wounds of WT mice from a post-filtered (cytokines, chemokines, growth factors and their respective receptors) pool of genes. Data are mean values ± SEM, statistical analysis: two-tailed Student's t test.

Suppl. Fig. 7

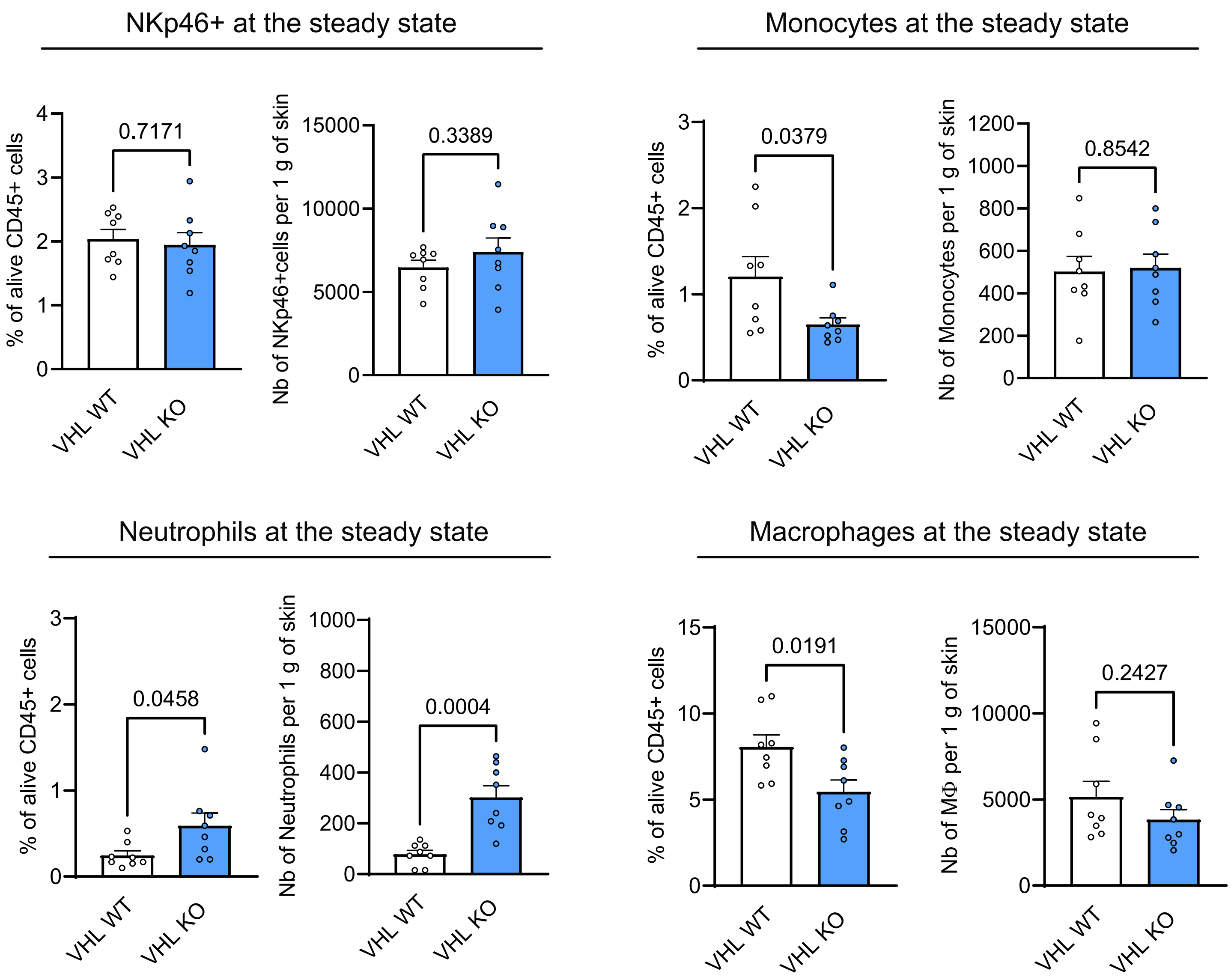
NKp46+ at the steady state
% of alive CD45+ cells
0.7171
Nb of NKp46+cells per 1 g of skin
0.3389
Monocytes at the steady state
% of alive CD45+ cells
0.0379
Nb of Monocytes per 1 g of skin
0.8542
Neutrophils at the steady state
% of alive CD45+ cells
0.0458
Nb of Neutrophils per 1 g of skin
0.0004
Macrophages at the steady state
% of alive CD45+ cells
0.0191
Nb of MΦ per 1 g of skin
0.2427
VHL WT
VHL KO

Supplementary figure 7. Constitutive activation of HIF-1α in NKp46$^+$ cells affects immune cell infiltration. Quantitative analysis by flow cytometry of NKp46$^+$ cells, neutrophils (CD11b$^+$, Ly6G$^+$), monocytes (CD11b$^+$, Ly6C$^+$) and macrophages (CD11b$^+$, Ly6C$^+$, F4/80$^+$), frequencies and absolute cell numbers per 1 gram of skin from WT and VHL KO mice, pooled data of 2 experiments (n=8). Data are mean values ± SEM, statistical analysis: two-tailed Student's t test.

Suppl. Fig. 8

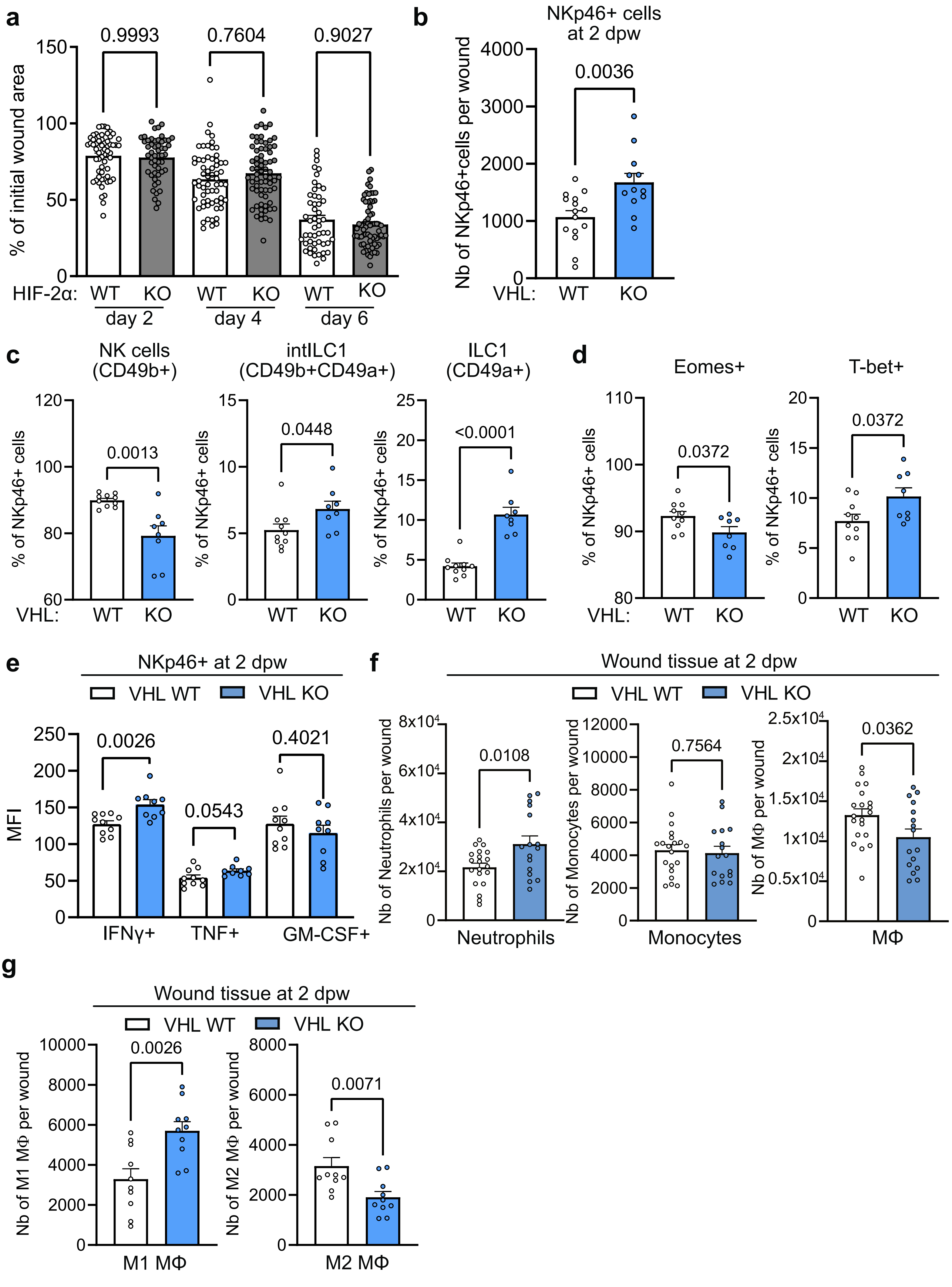
a
% of initial wound area
0.9993
0.7604
0.9027
HIF-2α: WT KO WT KO WT KO
day 2
day 4
day 6
b
NKp46+ cells at 2 dpw
Nb of NKp46+cells per wound
0.0036
VHL: WT KO
c
NK cells (CD49b+)
intILC1 (CD49b+CD49a+)
ILC1 (CD49a+)
% of NKp46+ cells
0.0013
0.0448
<0.0001
VHL: WT KO
d
Eomes+
T-bet+
0.0372
0.0372
e
NKp46+ at 2 dpw
VHL WT
VHL KO
MFI
0.0026
0.0543
0.4021
IFNγ+
TNF+
GM-CSF+
f
Wound tissue at 2 dpw
Nb of Neutrophils per wound
Nb of Monocytes per wound
Nb of MΦ per wound
0.0108
0.7564
0.0362
Neutrophils
Monocytes
MΦ
g
Wound tissue at 2 dpw
Nb of M1 MΦ per wound
Nb of M2 MΦ per wound
0.0026
0.0071
M1 MΦ
M2 MΦ

Supplementary figure 8. Loss of VHL in NKp46$^+$ cells changes ILC and myeloid cell composition in wounds. a Quantification of individual wound areas at 2, 4 and 6 days post wounding (dpw) in WT and HIF-2α KO animals relative to initial size at day 0, pooled data of 4 experiments, (n > 50), one-way ANOVA test. **b** Quantitative analysis of NKp46$^+$ cells, absolute numbers in skin wounds at 2 dpw, pooled data of 4 experiments. (n=15 for WT and n=12 for VHL KO). **c** Flow cytometry analysis for NK cells (NKp46$^+$, CD49b$^+$), intermediate ILC1 cells (NKp46$^+$, CD49b$^+$, CD49a$^+$) and ILC1 cells (NKp46$^+$, CD49a$^+$, CD49b$^-$) in skin wounds at 2 dpw, pooled data of 3 experiments, (n=10 for WT and n=8 for VHL KO). **d** Flow cytometry analysis for NKp46$^+$, NK1.1$^+$, Eomes$^+$ (NK cells) and NKp46$^+$, NK1.1$^+$, T-bet$^+$ (ILC1) cell populations in skin wounds at 2 dpw, pooled data of 3 different experiments (n=10 for WT and n=8 for VHL KO). **e** Flow cytometry analysis of mean fluorescence intensity (MFI) of IFNγ, TNFα and GM-CSF in NKp46$^+$, NK1.1$^+$ cells in skin wounds at 2 dpw, pooled data of 3 experiments (n=10 for WT and n=9 for VHL KO). **f** Flow cytometry analysis for neutrophils (CD11b$^+$, Ly6G$^+$), monocytes (CD11b$^+$, Ly6C$^+$) and macrophages (CD11b$^+$, Ly6C$^+$, F4/80$^+$), absolute numbers in skin wounds at 2 dpw, pooled data of 4 experiments. (n=20 for WT and n=16 for VHL KO). **g** Flow cytometry analysis for M1 (CD80$^+$, MHCII$^+$) and M2 macrophages (CD206$^+$), absolute numbers in skin wounds at 2 dpw, pooled data of 3 experiments (n=10). Data are mean values ± SEM, statistical analysis: two-tailed Student's t test unless stated otherwise.

Suppl. Fig. 9

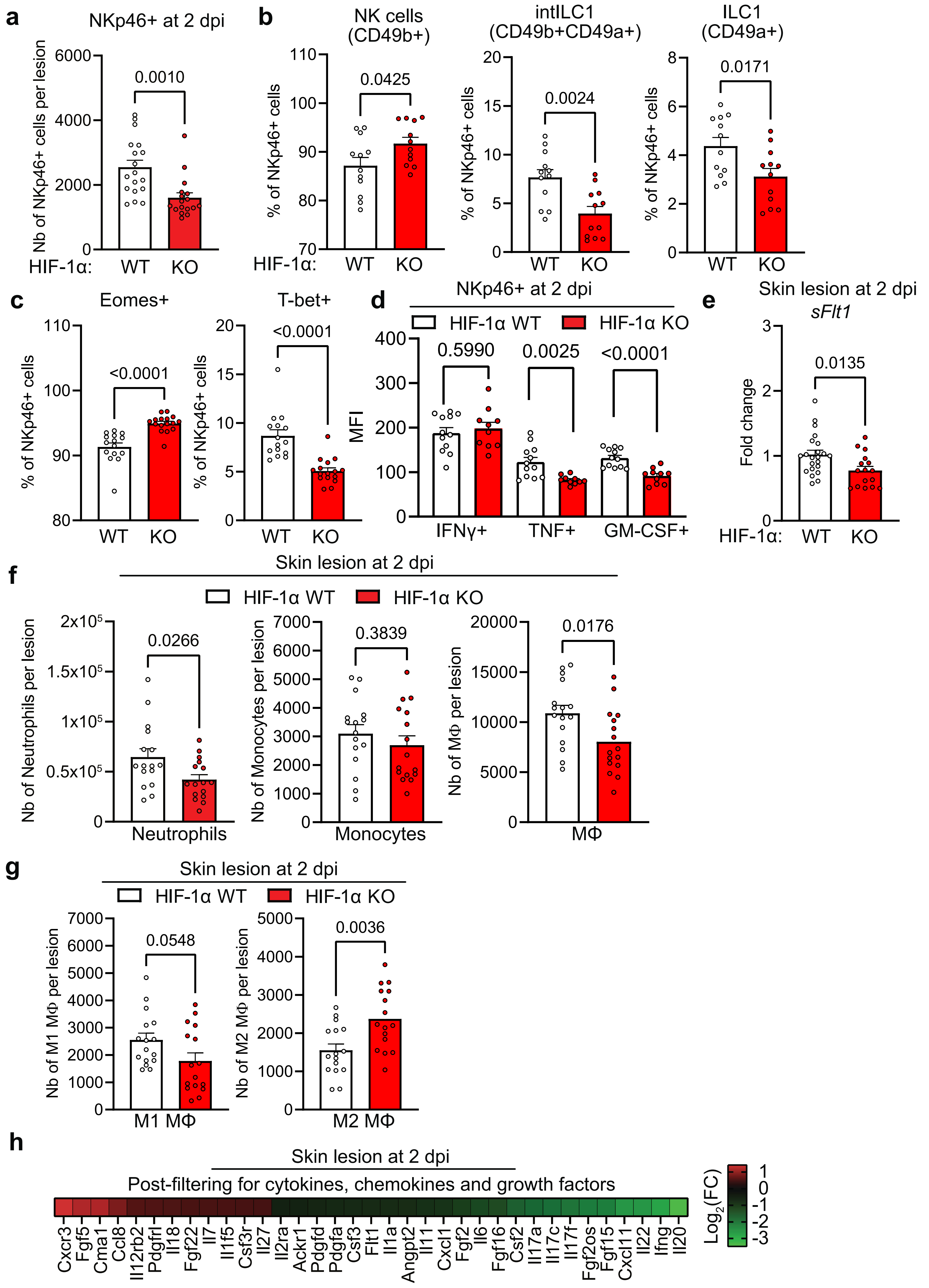
a
NKp46+ at 2 dpi
Nb of NKp46+ cells per lesion
6000
4000
2000
0
0.0010
HIF-1α:
WT
KO
b
NK cells (CD49b+)
% of NKp46+ cells
110
100
90
80
70
0.0425
HIF-1α:
WT
KO
intILC1 (CD49b+CD49a+)
% of NKp46+ cells
20
15
10
5
0
0.0024
WT
KO
ILC1 (CD49a+)
% of NKp46+ cells
8
6
4
2
0
0.0171
WT
KO
c
Eomes+
% of NKp46+ cells
110
100
90
80
<0.0001
WT
KO
T-bet+
% of NKp46+ cells
20
15
10
5
0
<0.0001
WT
KO
d
NKp46+ at 2 dpi
HIF-1α WT
HIF-1α KO
MFI
400
300
200
100
0
0.5990
0.0025
<0.0001
IFNγ+
TNF+
GM-CSF+
e
Skin lesion at 2 dpi
sFlt1
Fold change
3
2
1
0
0.0135
HIF-1α:
WT
KO
f
Skin lesion at 2 dpi
HIF-1α WT
HIF-1α KO
Nb of Neutrophils per lesion
2x10^5
1.5x10^5
1x10^5
0.5x10^5
0
0.0266
Neutrophils
Nb of Monocytes per lesion
7000
6000
5000
4000
3000
2000
1000
0
0.3839
Monocytes
Nb of MΦ per lesion
20000
15000
10000
5000
0
0.0176
MΦ
g
Skin lesion at 2 dpi
HIF-1α WT
HIF-1α KO
Nb of M1 MΦ per lesion
7000
6000
5000
4000
3000
2000
1000
0
0.0548
M1 MΦ
Nb of M2 MΦ per lesion
5000
4000
3000
2000
1000
0
0.0036
M2 MΦ
h
Skin lesion at 2 dpi
Post-filtering for cytokines, chemokines and growth factors
Cxcr3
Fgf5
Cma1
Ccl8
Il12rb2
Pdgfrl
Il18
Fgf22
Il7
Il1f5
Csf3r
Il27
Il2ra
Ackr1
Pdgfd
Pdgfa
Csf3
Flt1
Il1a
Angpt2
Il11
Cxcl1
Fgf2
Il6
Fgf16
Csf2
Il17a
Il17c
Il17f
Fgf2os
Fgf15
Cxcl11
Il22
Ifng
Il20
Log2(FC)
1
0
-1
-2
-3

Supplementary figure 9. HIF-1α in NKp46$^+$ cells supports the antimicrobial defence in the skin. a FACS analysis of NKp46$^+$ cells, absolute number in skin lesions at 2 days post infection (dpi), pooled data of 4 experiments, (n=18 for WT and n=17 for HIF-1α KO). **b** Flow cytometry analysis for NK cells (NKp46$^+$, NK1.1$^+$, CD49b$^+$), intermediate ILC1 cells (NKp46$^+$, NK1.1$^+$, CD49b$^+$, CD49a$^+$) and ILC1 cells (NKp46$^+$, NK1.1$^+$, CD49a$^+$, CD49b$^-$) in skin lesions at 2 dpi, pooled data of 3 experiments (n=12 for WT and HIF-1α KO mice). **c** Flow cytometry analysis for NKp46$^+$, NK1.1$^+$, Eomes$^+$ (NK cells) and NKp46$^+$, NK1.1$^+$, T-bet$^+$ (ILC1) cell populations in skin lesions at 2 dpi, pooled data of 4 experiments (n=15 for WT and n=16 for HIF-1α KO). d Flow cytometry analysis of mean fluorescence intensity (MFI) of IFNγ, TNF and GM-CSF in NKp46$^+$, NK1.1$^+$ cells in skin lesions at 2 dpi, pool data of 3 experiments (n=12 for WT and n=10 for HIF-1α KO). **e** Gene expression analysis of *sFflt1* in skin lesions at 2 dpi, pooled of 4 experiments (n=22 for WT and n=16 for HIF-1α KO). **f** Flow cytometry analysis for neutrophils (CD11b$^+$, Ly6G$^+$), monocytes (CD11b$^+$, Ly6C$^+$) and macrophages (CD11b$^+$, Ly6C$^+$, F4/80$^+$), absolute numbers in skin lesions at 2 dpi, pooled data of 4 experiments (n=16 for WT and HIF-1α KO mice). **g** Flow cytometry analysis for M1 (CD80$^+$, MHCII$^+$) and M2 macrophages (CD206$^+$), absolute numbers in skin lesions at 2 dpi, pooled data of 4 experiments (n=16 for WT and HIF-1α KO mice). **h** Heat map of gene expression in GAS skin lesions from HIF-1α KO mice with a significantly differential expression relative to GAS skin lesions from WT mice from a post-filtered (cytokines, chemokines, growth factors and their respective receptors) pool of genes. Data are mean values ± SEM, statistical analysis: two-tailed Student's t test.

Supp. Fig. 10

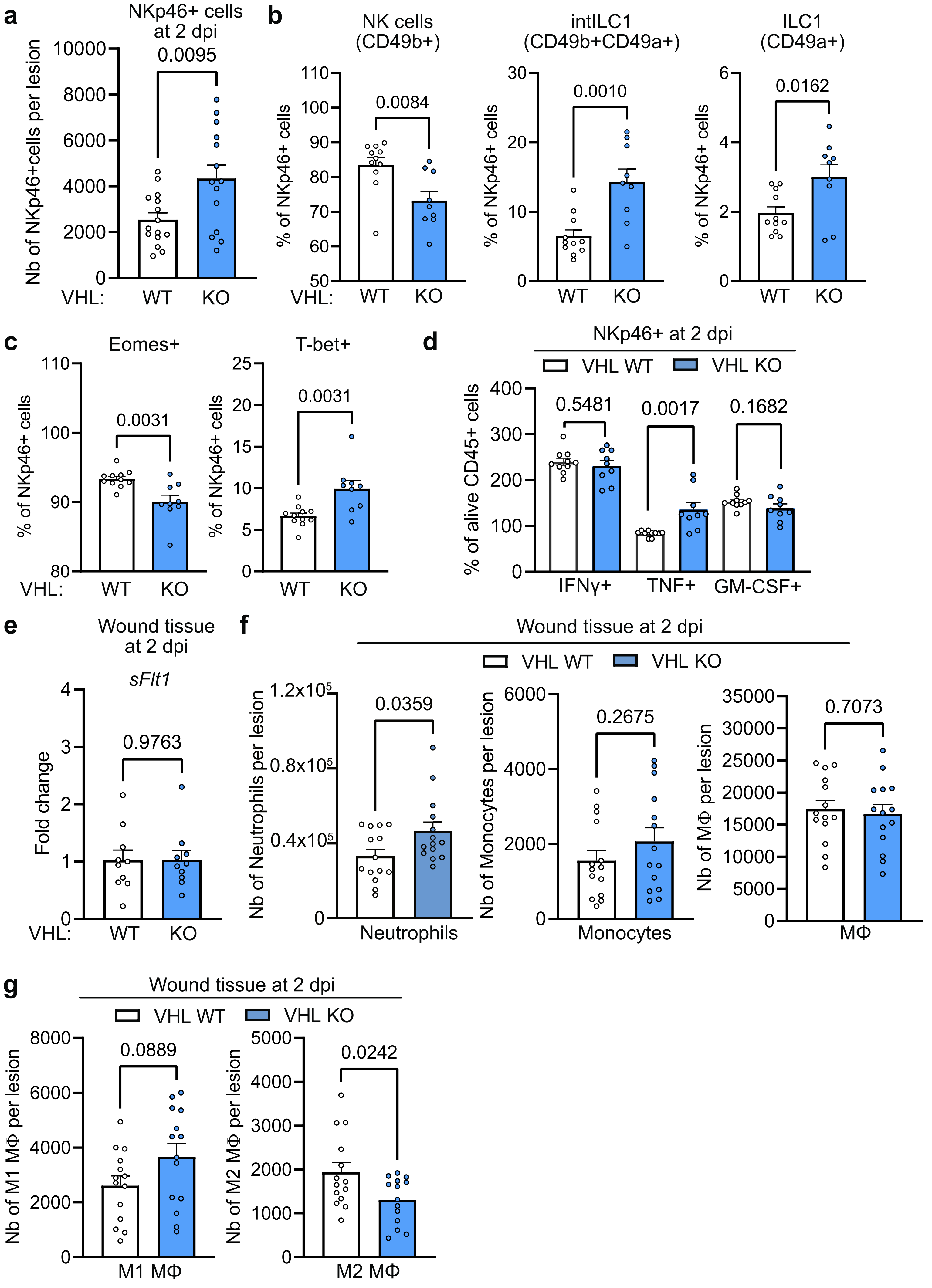
a
NKp46+ cells at 2 dpi
Nb of NKp46+cells per lesion
0.0095
VHL: WT KO
b
NK cells (CD49b+)
intILC1 (CD49b+CD49a+)
ILC1 (CD49a+)
% of NKp46+ cells
0.0084
0.0010
0.0162
c
Eomes+
T-bet+
0.0031
0.0031
d
NKp46+ at 2 dpi
VHL WT
VHL KO
% of alive CD45+ cells
0.5481
0.0017
0.1682
IFNγ+
TNF+
GM-CSF+
e
Wound tissue at 2 dpi
sFlt1
Fold change
0.9763
f
Wound tissue at 2 dpi
Nb of Neutrophils per lesion
0.0359
Neutrophils
Nb of Monocytes per lesion
0.2675
Monocytes
Nb of MΦ per lesion
0.7073
MΦ
g
Wound tissue at 2 dpi
Nb of M1 MΦ per lesion
0.0889
M1 MΦ
Nb of M2 MΦ per lesion
0.0242
M2 MΦ

Supplementary figure 10. Loss of VHL in NK cells protects against bacterial skin infections. a Quantitative analysis of NKp46$^+$ cells, absolute numbers in skin lesions at 2 days post infection (dpi), pooled of 4 experiments (n=15 for WT and n=14 for VHL KO). **b** Flow cytometry analysis for NK cells (NKp46$^+$, NK1.1$^+$, CD49b$^+$), intermediate ILC1 cells (NKp46$^+$, NK1.1$^+$, CD49b$^+$, CD49a$^+$) and ILC1 cells (NKp46$^+$, NK1.1$^+$, CD49a$^+$, CD49b$^-$) in skin lesions at 2 dpi, pooled data of 3 experiments (n=11 for WT and n=9 for VHL KO). **c** Flow cytometry analysis for NKp46$^+$, NK1.1$^+$, Eomes$^+$ (NK cells) and NKp46$^+$, NK1.1$^+$, T-bet$^+$ (ILC1) cell populations in skin lesions at 2 dpi, pooled data of 3 experiments (n=11 for WT and n=9 for VHL KO). **d** Flow cytometry analysis of mean fluorescence intensity (MFI) of IFNγ, TNF and GM-CSF in NKp46$^+$, NK1.1$^+$ cells in skin lesions at 2 dpi, pooled data of 3 experiments. (n=10 for WT and n=9 for VHL KO). **e** Gene expression analysis of *sFflt1* in skin lesions at 2 dpi, pooled data of 3 experiments (n=10 for WT and VHL KO mice). **f** Flow cytometry analysis for neutrophils (CD11b$^+$, Ly6G$^+$), monocytes (CD11b$^+$, Ly6C$^+$) and macrophages (CD11b$^+$, Ly6C$^+$, F4/80$^+$), absolute numbers in skin lesions at 2 dpi, pooled data of 4 experiments (n=14 for WT and VHL KO mice). **g** Flow cytometry analysis for M1 (CD80$^+$, MHCII$^+$) and M2 macrophages (CD206$^+$) absolute numbers in skin lesions at 2 dpi, pool data of 4 different experiments (n=14 for WT and VHL KO mice). Data are mean values ± SEM, statistical analysis: two-tailed Student's t test.

Supplementary Table 1. List of antibodies

	Antibody	Clone	Referance	Supplier	Dilution	Host Species
1	anti-F4/80	BM8	123107; 123131	BioLegend	1/100	Rat
2	anti-CD11c	N418	117310; 117306	BioLegend	1/100	Armenian Hamster
3	anti-CD64	X54-5/7.1	139307	BioLegend	1/100	Mouse
4	anti-CD8	53-6.7	100730	BioLegend	1/100	Rat
5	anti-CD206	C068C2	141732	BioLegend	1/100	Rat
6	anti-CD19	6D5	115530; 152404	BioLegend	1/100	Rat
7	anti-MHCII	M5/114.15.2	48-5321; 107616	eBioscience, BioLegend	1/100	Rat
8	anti-Ly6G	1A8	746448; 127618	BD, BioLegend	1/100	Rat
9	anti-Ly6C	HK1.4	128035	BioLegend	1/100	Rat
10	anti-CD80	16-10A1	104707	BioLegend	1/100	Armenian Hamster
11	anti-CD45	30-F11	564225; 103128	BD, BioLegend	1/100	Rat
12	anti-CD4	GK1.5	565974; 564667	BD	1/100	Rat
13	anti-B220	RA3-6B2	564662	BD	1/100	Rat
14	anti-CD11b	M1/70	564443; 101206	BD, BioLegend	1/100	Rat
15	anti-NK1.1	PK136	553165; 563220; 56[illegible]	BD	1/50	Mouse
16	anti-SiglecF	E50-2440	562757	BD	1/100	Rat
17	anti-TCRβ	H57-597	109210; 109206	BioLegend	1/100	Armenian Hamster
18	anti-TCRγδ	GL3	118106	BioLegend	1/100	Armenian Hamster
19	anti-NKp46	29A1.4	25-3351	eBioscience	1/50	Rat
20	anti-TER119/Erythroid cells	TER-119	116206	BioLegend	1/100	Rat
21	anti-CD127	A7R34	135027	BioLegend	1/100	Rat
22	anti-CCR6	140706	747831	BD	1/100	Rat
23	anti-CD49b	DX5	741752	BD	1/100	Armenian Hamster
24	anti-CD49a	Ha31/8	741976	BD	1/100	Armenian Hamster
25	anti-ICOS	C398.4A	15-9949-82	eBioscience	1/100	Armenian Hamster
26	anti-IFN-γ	XMG1.2	566151	BD	1/100	Rat
27	anti-TNF	MP6-XT22	506333	BD	1/100	Rat
28	anti-GM-CSF	MP1-22E9	554406	BD	1/100	Rat
29	anti-RORγt	Q31-378	562682	BD	1/100	Mouse
30	anti-T-bet	4b10	644835	BD	1/100	Mouse
31	anti-GATA3	TWAJ	12-9966-42	eBioscience	1/100	Rat
32	anti-Eomes	Dan11mag	61-4875-82	eBioscience	1/100	Rat

Supplementary Table 2. qRT-PCR Primer sequences (mouse)

Name of gene		Sequence
16s	forward primer	5′-AGATGATCGAGCCGCGC -3′
	reverse primer	5′-GCTACCAGGGCCTTTGAGATGGA-3′
Ifng	forward primer	5′- TCAAGTGGCATAGATGTGGAAGAA -3′
	reverse primer	5′- TGGCTCTGCAGGATTTTCATG -3′
Tnfa	forward primer	5′- GCCTCTTCTCATTCCTGCTTG -3′
	reverse primer	5′- CTGATGAGAGGGAGGCCATT -3′
Nos2	forward primer	5′- GCTTGGGTCTTGTTCACTCC -3′
	reverse primer	5′- GCAAGTGAAATCCGATGTGGC -3′
Arg1	forward primer	5′- AATCTGCATGGGCAACCTGT -3′
	reverse primer	5′- AGGGTCTACGTCTCGCAAG -3′
sFlt1	forward primer	5′- GTCACAGATGTGCCGAATGG -3′
	reverse primer	5′- TGACTTTGTGTGGTACAATC -3′
Il10	forward primer	5′- CCAGTTTTACCTGGTAGAGGTGATG -3′
	reverse primer	5′- TGTCTAGGTCCTGGAGTCCAGCAGACTC -3′
Csf2	forward primer	5′- TGGGCATTGTGGTCTACAGC -3′
	reverse primer	5′- GCGGGTCTGCACACATGTTA -3′
