# Supplementary material for: NK cells in hypoxic skin mediate a trade-off between wound healing and antibacterial defence
Source: Nat Commun. 2021 Aug 4;12:4700. doi: 10.1038/s41467-021-25065-w (PMC8338923; doi:10.1038/s41467-021-25065-w)
Supplement: Supplementary file 2 — Reporting Summary [file 41467_2021_25065_MOESM2_ESM.pdf]

nature portfolio

Corresponding author(s): Christian Stockmann

Last updated by author(s): Jun 17, 2021

# Reporting Summary

Nature Portfolio wishes to improve the reproducibility of the work that we publish. This form provides structure for consistency and transparency in reporting. For further information on Nature Portfolio policies, see our Editorial Policies and the Editorial Policy Checklist.

## Statistics

For all statistical analyses, confirm that the following items are present in the figure legend, table legend, main text, or Methods section.

| n/a | Confirmed | |
|---|---|---|
| ☐ | ☒ | The exact sample size (*n*) for each experimental group/condition, given as a discrete number and unit of measurement |
| ☐ | ☒ | A statement on whether measurements were taken from distinct samples or whether the same sample was measured repeatedly |
| ☐ | ☒ | The statistical test(s) used AND whether they are one- or two-sided<br>*Only common tests should be described solely by name; describe more complex techniques in the Methods section.* |
| ☐ | ☒ | A description of all covariates tested |
| ☐ | ☒ | A description of any assumptions or corrections, such as tests of normality and adjustment for multiple comparisons |
| ☐ | ☒ | A full description of the statistical parameters including central tendency (e.g. means) or other basic estimates (e.g. regression coefficient) AND variation (e.g. standard deviation) or associated estimates of uncertainty (e.g. confidence intervals) |
| ☐ | ☒ | For null hypothesis testing, the test statistic (e.g. *F*, *t*, *r*) with confidence intervals, effect sizes, degrees of freedom and *P* value noted<br>*Give P values as exact values whenever suitable.* |
| ☒ | ☐ | For Bayesian analysis, information on the choice of priors and Markov chain Monte Carlo settings |
| ☒ | ☐ | For hierarchical and complex designs, identification of the appropriate level for tests and full reporting of outcomes |
| ☒ | ☐ | Estimates of effect sizes (e.g. Cohen's *d*, Pearson's *r*), indicating how they were calculated |

*Our web collection on statistics for biologists contains articles on many of the points above.*

## Software and code

Policy information about availability of computer code

| | |
|---|---|
| Data collection | Flow cytometry was carried out on a BD FACSymphony A5 Flow Cytometer. Images were acquired using Leica DM 6000 or Nikon Eclipse Ti. qRT-PCR analysis was performed on LightCycler 96 (Roche). |
| Data analysis | Gene ontology network analysis was performed with ClueGO 2.5.8/Cytoscape 3.<br>Image analysis and quantifications were performed using ImageJ 1.49 (National Institutes of Health, USA) and ZEN 3.3 Blue edition (Zeiss) imaging software.<br>Flowcytometry data were aquired using BD FACSDiva 9.0 (BD) and analysed using FlowJo v10 (Treestar). Statistical analysis was performed with the Prism 8.3 software (GraphPad Software). |

For manuscripts utilizing custom algorithms or software that are central to the research but not yet described in published literature, software must be made available to editors and reviewers. We strongly encourage code deposition in a community repository (e.g. GitHub). See the Nature Portfolio guidelines for submitting code & software for further information.

## Data

Policy information about availability of data

All manuscripts must include a data availability statement. This statement should provide the following information, where applicable:

- Accession codes, unique identifiers, or web links for publicly available datasets
- A description of any restrictions on data availability
- For clinical datasets or third party data, please ensure that the statement adheres to our policy

Source data are provided with this paper (doi: 10.17632/x3mmvmy9sg.1).
The differentially expressed genes from RNAseq of wound healing study have been deposited in the Mendeley database under doi: 10.17632/3xkcccj9n4.1

The differentially expressed genes from RNAseq of group A Streptoccocus infection study have been deposited in the Mendeley database under doi: 10.17632/7nk8j9h6yp.1

# Field-specific reporting

Please select the one below that is the best fit for your research. If you are not sure, read the appropriate sections before making your selection.

- [x] Life sciences
- [ ] Behavioural & social sciences
- [ ] Ecological, evolutionary & environmental sciences

For a reference copy of the document with all sections, see nature.com/documents/nr-reporting-summary-flat.pdf

# Life sciences study design

All studies must disclose on these points even when the disclosure is negative.

| | |
|---|---|
| Sample size | No sample-size calculation was performed. The sample size was determined to detect significant changes. At least three mice per genotype per experiment were used. |
| Data exclusions | The genes expression data were analyzed with the ROUT method (Q=1%) to identify outliers. Outliers were excluded from the analyses. |
| Replication | Majority of experiments reported in this study were repeated at least four independent times, some repeated two or three. All information about number of experimental repeats are stated in legends. All samples evaluated at the level of RNAseq were performed in triplicate and the sequencing was all successful. |
| Randomization | Littermates of the same genotype were randomly assigned to experimental groups. Animals were used between 8 and 12 weeks of age. |
| Blinding | The investigators were blinded to group allocation during data collection and analysis. |

# Reporting for specific materials, systems and methods

We require information from authors about some types of materials, experimental systems and methods used in many studies. Here, indicate whether each material, system or method listed is relevant to your study. If you are not sure if a list item applies to your research, read the appropriate section before selecting a response.

## Materials & experimental systems

| n/a | Involved in the study | |
|---|---|---|
| [ ] | [x] | Antibodies |
| [ ] | [x] | Eukaryotic cell lines |
| [x] | [ ] | Palaeontology and archaeology |
| [ ] | [x] | Animals and other organisms |
| [x] | [ ] | Human research participants |
| [x] | [ ] | Clinical data |
| [x] | [ ] | Dual use research of concern |

## Methods

| n/a | Involved in the study | |
|---|---|---|
| [x] | [ ] | ChIP-seq |
| [ ] | [x] | Flow cytometry |
| [x] | [ ] | MRI-based neuroimaging |

## Antibodies

Antibodies used

Flow cytometry:
The following mAbs from eBioscience or BD-Biosciences or BioLegend were used: anti-F4/80 (BM8; 123107; 123131), anti-CD11c (N418; 117310;117306), anti-CD64 (X54-5/7.1; 139307), anti-CD8 (53-6.7; 100730), anti-CD206 (C068C2; 141732), anti-CD19 (6D5; 115530; 152404), anti-MHCII (M5/114.15.2; 48-5321; 107616), anti-Ly6G (1A8; 746448; 127618), anti-Ly6C (HK1.4; 128035), anti-CD80 (16-10A1; 104707), anti-CD45 (30-F11; 564225; 103128), anti-CD4 (GK1.5; 565974; 564667), anti-B220 (RA3-6B2; 564662), anti-CD11b (M1/70; 564443; 101206), anti-NK1.1 (PK136; 553165; 563220; 564144), anti-Siglec-F (E50-2440; 562757), anti-TCRβ (H57-597; 109210; 109206), anti-TCRγδ (GL3; 118106), anti-NKp46 (29A1.4; 25-3351), anti-TER119/Erythroid cells (TER-119; 116206), anti-CD127 (A7R34; 135027), anti-CCR6 (140706; 747831), anti-CD49b (DX5; 741752), anti-CD49a (Ha31/8; 741976), anti-ICOS (C398.4A; 15-9949-82) and relevant isotype controls.
anti-IFN-γ (XMG1.2; BD, 566151), anti-TNF-α (MP6-XT22; BD, 506333), anti-GM-CSF (MP1-22E9; BD, 554406).
anti-RORγt (Q31-378; BD, 562682), anti-T-bet (4b10; BD, 644835), anti-GATA3 (TWAJ; eBioscience, 12-9966-42), and anti-Eomes (Dan11mag; eBioscience, 61-4875-82)
Primary antibodies used for immmunohistochemistry and immunofluorescence:
(a) rat anti-CD31 at 1:10 dilution (DIANOVA; DIA-310)
(b) goat anti-NKp46 at 3µg/mL dilution (R&D; AF2225).
The fluorochrome-conjugated Alexa 488 (ThermoFisher, A11055) and DyLight 594 (ThermoFisher, SA5-10028) were used as secondary antibodies (1:200).

| | |
|---|---|
| Validation | All of the listed antibodies used for FACS have been validated by the manufacturer by flow cytometry (as indicated in the manufacturers' website). In addition, to avoid off-target binding, antibodies were titrated before using them in experimental work. The background binding was verified relying on the isotype controls. |

# Eukaryotic cell lines

Policy information about cell lines

| | |
|---|---|
| Cell line source(s) | Mouse endothelial cells (mECs) were kindly provided by prof. Veronika Sexl (Kollmann, K. et al. A kinase-independent function of CDK6 links the cell cycle to tumor angiogenesis. Cancer Cell 24, 167–181 (2013).) |
| Authentication | Expression of the endothelial cell-specific marker CD31 was verified by immunocytochemistry using an anti-CD31 antibody from (Dianova, DIA-310). |
| Mycoplasma contamination | Cell line was tested negative. |
| Commonly misidentified lines (See ICLAC register) | No commonly misidentified cell line was used in this study. |

# Animals and other organisms

Policy information about studies involving animals; ARRIVE guidelines recommended for reporting animal research

| | |
|---|---|
| Laboratory animals | Mouse models<br>Targeted deletion of HIF-1!, and VHL in NKp46-expressing NK cells was achieved by crossing the loxP-flanked HIF-1! allele17, the loxP-flanked VHL allele24 or loxP-flanked EPAS1 allele25 to the Ncr1 (NKp46) promoter-driven cre recombinase (termed HIF-1! KO mice, VHL KO mice or HIF-2! KO mice). Cre recombinase negative mice from following strains were used as HIF-1! WT mice or VHL WT mice. To mitigate the influence of strain variation, mice were kept in a >99% C57Bl/6J background. Mouse experiments were performed with at least three mice per group and multiple experiments were combined to assess statistically significant differences as noted. Littermates of the same genotype were randomly assigned to experimental groups. Animals were used between 8 and 12 weeks of age. Mice were housed at facility following requirments for ABSL-2 at ambient temperature and humidity on a 12h light cycle. Not more than 5 mice or less than 2 mice were housed together. Animals were allowed free excess to feed and water ad libitum. Male mice were used for wound healing model, while for GAS infection model male and females were used. |
| Wild animals | The study did not involve wild animals |
| Field-collected samples | The study did not involve sample collection from the field. |
| Ethics oversight | All animal experiments have been approved by the veterinary authorities of Canton of Zurich, Switzerland (ZH 219/2017, ZH 169/2018), and were performed in accordance with Swiss law on the care, welfare, and treatment of animals. |

Note that full information on the approval of the study protocol must also be provided in the manuscript.

# Flow Cytometry

## Plots

Confirm that:

- [x] The axis labels state the marker and fluorochrome used (e.g. CD4-FITC).
- [x] The axis scales are clearly visible. Include numbers along axes only for bottom left plot of group (a 'group' is an analysis of identical markers).
- [x] All plots are contour plots with outliers or pseudocolor plots.
- [x] A numerical value for number of cells or percentage (with statistics) is provided.

## Methodology

| | |
|---|---|
| Sample preparation | Tissues were placed in gentleMACS C Tubes (Miltenyi) with 5 ml of HBSS/HEPES (w/ Ca2+, w/ Mg2+, 10 mM HEPES). Next, tissues were cut into small pieces in size of 1-2 mm2. Then, HBSS/HEPES was supplemented with 2U/ml of Dispase II (Roche), 0.55 WU/ml of Liberase TL (Roche) and 200 KU/ml of DNAseI (Roche) and digestion reaction was performed at 37°C with continuous shaking for 1 hour. At the end of incubation, C Tubes were attached upside down onto the sleeve of the gentleMACS Dissociator and run twice for program D. Cell suspensions were filtered through 70 μm CellStrainer and cells were washed twice with HBSS (w/o Ca2+, w/o Mg2+) buffer containing 2%FBS. Single-cell suspension of skin wounds and skin lesions were stained with respective mAbs from eBioscience or BD-Biosciences or BioLegend. Cell viability was measured using LIVE/DEAD® Fixable Aqua Dead Cell Stain Kit (Thermo Fisher). |
| Instrument | BD FACSymphony™ A5 Flow Cytometer |
| Software | FlowJo v10 (Treestar) and Diva 9.0 (BD) |

| | |
|---|---|
| Cell population abundance | NKp46+: 2-4% in CD45+ cells |
| Gating strategy | Single cells suspension from skin wounds were gated on NKp46+ cells in the alive CD45+ cell population. Subsequent gating on:<br>ILC1 group cells (NKp46+, NK1.1+) and ILC3 cells (NKp46+, NK1.1-, RORγt+)<br>or<br>NK cells (NKp46+, CD49b+), intermediate ILC1cells (NKp46+, CD49b+, CD49a+) and ILC1cells (NKp46+, CD49a+, CD49b-) in the NKp46+ cell population<br>or<br>for neutrophils (CDllb+, Ly6G+), monocytes (CDllb+, Ly6C+), macrophages (CDllb+, Ly6C+, F4/80+) and M2 macrophages frequency (CD206+) to Ml macrophages frequency (CD80+)<br>or<br>frequency of IFNγ, TNFa and GM-CSF expressing NKp46+ cells and mean fluorescence intensity (MFI) of IFNγ, TNFa and GM-on NKp46+ cells. |

☒ Tick this box to confirm that a figure exemplifying the gating strategy is provided in the Supplementary Information.
